# Rumen Fluid Metabolomics and Microbiome Profiling of Dairy Cows Fed Combinations of Prebiotics, Essential Oil Blend, and Onion Peel Using the RUSITEC System

**DOI:** 10.3390/metabo15120762

**Published:** 2025-11-25

**Authors:** Joel O. Alabi, Ahmed E. Kholif, Kelechi A. Ike, Deborah O. Okedoyin, Oludotun O. Adelusi, Michael Wuaku, Chika C. Anotaenwere, James M. Enikuomehin, Olatunde A. Oderinwale, John O. Adebayo, Andrea R. Gentry-Apple, Uchenna Y. Anele

**Affiliations:** 1 Department of Animal Sciences, North Carolina Agricultural and Technical State University, Greensboro, NC 27411, USA; joalabi@aggies.ncat.edu (J.O.A.); aekholif@ncat.edu (A.E.K.); kaike@aggies.ncat.edu (K.A.I.); dookedoyin@aggies.ncat.edu (D.O.O.); ooadelusi@aggies.ncat.edu (O.O.A.); mwuaku@aggies.ncat.edu (M.W.); ccanotaenwere@aggies.ncat.edu (C.C.A.); jmenikuomehin@aggies.ncat.edu (J.M.E.); oaoderinwale@aggies.ncat.edu (O.A.O.); joadebayo@aggies.ncat.edu (J.O.A.); argentry@ncat.edu (A.R.G.-A.); 2 Dairy Science Department, National Research Centre, 33 Bohouth St. Dokki, Giza 12622, Egypt

**Keywords:** dairy cows, dysbiosis, feed additives, metabolome, microbiota, RUSITEC

## Abstract

Background and Objectives: Dairy products provide vital energy, high-quality protein, and micronutrients for over six billion people worldwide, with dairy cows contributing nearly 81% of global milk production. Sustainable strategies to enhance productivity are therefore critical. Feed additives such as essential oil blends (EOB), onion peel (OPE), and prebiotics including mannan oligosaccharides (MOS) and galacto-oligosaccharides (GOS) have been proposed to improve rumen fermentation, modulate microbial ecology, and mitigate greenhouse gas emissions. This study evaluated the combined effects of EOB, OPE, MOS, and GOS on rumen metabolism using the rumen simulation technique (RUSITEC). Materials and Methods: Rumen inoculum from three cannulated Holstein Friesian cows was incubated across 16 vessels (four treatments × four replicates) for nine days. Treatments included a control (CON; TMR only), GEO (TMR + GOS + EOB + OPE), MEO (TMR + MOS + EOB + OPE), and OLEO (TMR + a 1:1 mixture of GOS and MOS + EOB + OPE). Additives were included at 3 µL/g TMR for EOB and 30 mg/g TMR (3% *w*/*w*) for OPE, GOS, MOS, or OLG. Rumen effluents were collected for untargeted metabolomic profiling by liquid chromatography–mass spectrometry, identifying 661 metabolites. Results: Partial least squares-discriminant analysis revealed clear separation between CON and additive groups, confirming distinct metabolic shifts. GEO primarily enhanced tryptophan, tyrosine, and purine metabolism; MEO stimulated phosphonate and pyrimidine pathways and bile acid biosynthesis; OLEO promoted phosphonate, nicotinamide, and taurine metabolism. Microbial analysis showed enrichment of taxa such as *Lachnospira*, *Succinivibrionaceae*, *Macellibacteroides*, *Lysinibacillus*, and *Christensenellaceae*, indicating complementary effects on fermentation and microbial stability. Conclusions: These results demonstrate that dietary supplementation with GEO, MEO, or OLEO modulates rumen metabolism and microbial ecology without impairing fermentation, supporting improved nutrient utilization, antioxidant defenses, and metabolic resilience in dairy cows, with potential benefits for productivity and sustainability.

## 1. Introduction

Dairy products play a pivotal role in global food security, supplying essential nutrients such as proteins, vitamins, minerals, and fatty acids to over six billion people worldwide. Dairy cows contribute approximately 81% of the world’s milk supply, with the United States ranking as the world’s second-largest producer. This underscores the need for sustainable intensification strategies to meet rising demand while minimizing environmental impacts. Dairy cattle production systems face the dual challenge of improving productivity and animal health while reducing reliance on antibiotics and mitigating greenhouse gas (GHG) emissions. Feed additives such as essential oil blends (EOB), onion peel extract (OPE), and prebiotics including mannan-oligosaccharides (MOS) and galacto-oligosaccharides (GOS) have received considerable attention as promising alternatives to antibiotics and synthetic growth promoters. These strategies align with the “One Health” concept, which emphasizes reducing antimicrobial resistance while supporting sustainable food production.

Essential oils contain a diverse array of bioactive compounds, such as terpenes, phenolic acids, and flavonoids, which exhibit potent antioxidant activities by scavenging free radicals, thus reducing oxidative stress and improving cattle health and productivity [1]. Furthermore, essential oils demonstrate strong antimicrobial activity against pathogenic bacteria, fungi, and viruses, while favoring beneficial ruminal microbes [2]. This ultimately promotes optimal digestion and nutrient utilization [3,4]. Beyond antimicrobial activity, essential oils have been linked to modulation of ruminal fermentation, reduction in methane (CH_4_) production, and improvement in feed efficiency, which are critical for sustainable livestock systems.

Prebiotics such as MOS, derived from the outer cell wall of *Saccharomyces cerevisiae*, are recognized for their stability during feed processing and for their role in enhancing livestock growth performance, modulating intestinal microflora, and strengthening immune function [5]. MOS has been shown to improve gastrointestinal and ruminal health, reduce enteric CH_4_ and carbon dioxide (CO_2_) emissions [6], and serve as a viable antibiotic alternative. In dairy systems, MOS supplementation has also been linked to improved colostrum quality, increased transfer of immunoglobulins, and enhanced calf health outcomes [7]. Similarly, GOS, a naturally occurring oligosaccharide and important component of milk, selectively promotes the proliferation of bifidobacteria and other beneficial microbes, leading to enhanced volatile fatty acid (VFA) production, improved gut epithelial integrity, and suppression of potentially pathogenic bacteria [8,9,10]. Notably, GOS supplementation has also been associated with reduced incidence of gastrointestinal disorders in young calves, highlighting its potential for improving both productivity and animal welfare.

Onion peel, a rich source of bioactive phytochemicals including quercetin, allicin, and kaempferol, has been reported to possess antimicrobial and antioxidant properties that can alter ruminal fermentation patterns, improve nutrient degradability, and inhibit methanogenic archaea activity, thereby reducing CH_4_ emissions [4,11,12]. Since OPE is an agricultural by-product, its use also supports circular economy principles by valorizing waste streams into functional feed ingredients. Taken together, these feed additives provide multiple avenues to improve dairy cow performance, enhance resilience against disease, and mitigate negative environmental impacts.

Recent attention has shifted toward the use of combinations of feed additives, with the rationale that they may exert combined effects on rumen fermentation, nutrient utilization, and environmental sustainability beyond what is achievable with individual compounds. Several studies have demonstrated promising outcomes when combining essential oils, prebiotics, and plant extracts in ruminant diets. For instance, Patra and Yu [13] showed that pairing essential oils such as eugenol and carvacrol with fumarate further enhanced CH_4_ reduction compared to single treatments, with essential oils directly suppressing methanogens while fumarate served as an alternative hydrogen sink. Similarly, Alabi et al. [14] reported that supplementing essential oil blends with fumaric acid in the RUSITEC system increased the molar proportion of propionate while lowering the acetate-to-propionate ratio. This combination reduced CH_4_ emissions by 60.2% without compromising dry matter (DM) disappearance, fiber digestibility, ruminal pH, or total gas production. Additional evidence [5] suggests that additivity between MOS and OPE improved substrate fermentation, resulting in greater gas and VFA production, enhanced energy efficiency, and reduced enteric CH_4_ emissions. The combination also enhanced fiber utilization by promoting the degradation of acid detergent fiber and acid detergent lignin.

Building upon these findings, the rationale for the present study was to formulate and evaluate specific combinations of feed additives capable of optimizing rumen fermentation efficiency while mitigating GHG emissions. Previous studies have investigated the individual effects of EOB, OPE, GOS, and MOS, as well as various additive combinations, including GOS + MOS, GOS + EOB, GOS + OPE, MOS + OPE, GOS + MOS + EOB, GOS + MOS + OPE (GMOP), and MOS + EOB [4]. Among these, EOB (200 µL/g) was found to lower CH_4_ emissions by 58%, while EOB, MOS + EOB, GOS + EOB, and GOS + MOS + EOB reduced CO_2_, ammonia, and hydrogen sulfide concentrations relative to the control. However, higher EOB inclusion levels were associated with reduced total and molar proportions of VFA, suggesting possible negative effects on fermentation efficiency. Consequently, the present study employed a lower EOB dosage (3 µL/g) in the formulations (GEO, MEO, and OLEO) and found that these specific combinations maintain fermentation performance with slight variation on VFA production [12]. Despite these encouraging findings, the mechanisms underlying these combined effects remain insufficiently understood, primarily due to the complexity of host–microbe–diet interactions in the rumen. Thus, integrative approaches combining microbial profiling with metabolomics are increasingly needed to unravel how feed additives modulate rumen ecology and function.

The rumen microbiota is a complex community of microorganisms that play a vital role in nutrient metabolism, immune regulation, and maintenance of host homeostasis [15,16]. Dysbiosis refers to an imbalance in the composition or function of these microbial populations, often characterized by reduced microbial diversity, overgrowth of pathogenic taxa, and depletion of beneficial species [17]. Such imbalances can disrupt metabolic pathways, leading to impaired fermentation, altered production of VFA, and accumulation of toxic metabolites [18,19]. In cattle, dysbiosis has been linked to metabolic disorders including obesity, insulin resistance, hepatic steatosis, and inflammatory conditions [20,21]. Emerging evidence also highlights its role in ruminal acidosis, impaired nutrient utilization, and systemic inflammation in livestock [18,19]. Therefore, maintaining a balanced rumen microbiome is a key determinant of animal productivity, health, and environmental performance.

Metabolomics, the comprehensive characterization of small molecules within biological systems, has emerged as a powerful analytical tool to decipher the biochemical consequences of dietary interventions. Non-targeted metabolomics using high-resolution platforms such as LC–MS, Orbitrap, and Fourier-transform ion cyclotron resonance enables broad detection of metabolites, while targeted metabolomics allows for highly sensitive quantification of specific compounds [22]. By capturing metabolite signatures associated with feed additives, metabolomics provides deeper insights into energy metabolism, microbial fermentation pathways, and nutrient utilization. Indeed, metabolomic profiling has revealed critical associations between diet composition, microbial community shifts, and host metabolic responses [23,24], and has been applied to study feed efficiency, health biomarkers, and GHG mitigation strategies in ruminants [25,26,27]. When integrated with microbiome profiling, metabolomics offers a holistic systems-biology framework to link microbial activity with host physiology and environmental outcomes.

16S rRNA gene sequencing is a widely used molecular technique for profiling microbial communities by targeting the highly conserved 16S ribosomal RNA gene in bacteria. In dairy cows, this approach enables detailed analysis of gut and rumen microbiota, offering insights into microbial diversity, function, and response to dietary interventions [4]. Such analyses are crucial for optimizing animal health, productivity, and feed efficiency. For example, Liu et al. [28] reported that feeding calves a mixture of oregano essential oil and yeast cultures led to increased rumen microbial richness and diversity, along with regulated relative abundances of particular species. Similarly, Chang et al. [29] reported that GOS supplementation enhanced microbial diversity, reduced incidence of diarrhea and improved growth performance of preweaning dairy calves. Nevertheless, studies investigating the rumen metabolome of dairy cows supplemented with EOB, OPE, MOS, and GOS remain limited. Additionally, through 16S ribosomal ribonucleic acid (rRNA) sequencing and microbiota profiling, the findings will support the development of targeted, sustainable feeding strategies that enhance cattle productivity while addressing environmental sustainability. Therefore, the present study aimed to investigate the combined effects of these feed additives on ruminal metabolite profiles, biochemical pathways and rumen microbiome using the rumen simulation technique (RUSITEC), thereby providing novel insights into their potential to improve dairy cattle productivity and environmental sustainability. We hypothesize that integrating metabolomic and microbiome profiling will reveal combined effects of EOB, OPE, MOS, and GOS on ruminal metabolic pathways and microbial networks, thereby identifying novel biochemical and microbial markers associated with improved dairy cattle productivity and environmental sustainability.

## 2. Materials and Methods

### 2.1. Study Approval

All experimental procedures were conducted in compliance with the standards established by the Institutional Animal Care and Use Committee (Protocol No. LA22-0019), North Carolina A&T State University, Greensboro. The dairy cows used in this study were maintained under University Farm welfare and husbandry guidelines, ensuring compliance with ethical standards for animal use in research.

### 2.2. Experimental Design

A total mixed ration (TMR) was formulated with corn silage, alfalfa hay, and concentrate at a 3:1:1 DM ratio, with nutrient composition previously described [12]. An EOB, consisting of garlic, lemongrass, cumin, lavender, and nutmeg in a 4:2:2:1:1 ratio, was selected based on prior evidence of CH_4_ reduction [14]. The study was conducted using the Rumen Simulation Technique (RUSITEC) system as outlined by Alabi et al. [14]. Sixteen 1-L fermentation vessels were randomly assigned to four treatments, each with four replicates. Each vessel was inoculated with 700 mL rumen fluid collected from three rumen-cannulated, non-lactating Holstein Friesian cows and 200 mL McDougall’s buffer [Rumen fluid was collected from three rumen-cannulated, non-lactating Holstein Friesian cows, pooled, and thoroughly mixed prior to inoculation to ensure a homogeneous microbial inoculum. The artificial saliva (McDougall’s buffer) was prepared according to the standard formulation, containing NaHCO_3_ (9.83 g/L), Na_2_HPO_4_ (3.69 g/L), KCl (0.60 g/L), NaCl (0.47 g/L), (NH_4_)_2_SO_4_ (0.30 g/L), MgCl_2_·6H_2_O (0.061 g/L), and CaCl_2_·2H_2_O (0.0293 g/L)]. In situ bags (70 × 140 mm, 150 μm pore size; Ankom, Macedon, NY, USA) containing 10 ± 0.2 g of substrate were incubated under anaerobic conditions for 48 h. Treatments included:

(1) CON: TMR only,
(2) GEO: TMR supplemented with GOS + EOB + OPE,
(3) MEO: TMR supplemented with MOS + EOB + OPE,
(4) OLEO: TMR supplemented with a combination of GOS and MOS (1:1, OLG) + EOB + OPE.

Additives were included at 3 µL/g TMR for EOB and 30 mg/g TMR (i.e., 3% *w*/*w*) for OPE, GOS, MOS, or OLG. The 9-day experimental period included a 3-day adaptation phase followed by 6 days of data collection, a design intended to stabilize microbial populations before sampling. The doses of the additives were determined based on findings from previous studies, as noted earlier [4,12].

### 2.3. Rumen Fluid Sampling and Liquid Chromatography-Mass Spectrometry (LC-MS) Metabolite Quantification

Rumen effluents (50 mL per vessel) were collected daily from all replicates within a treatment group. On each sampling day (d 4–9), samples obtained from the four vessels within each treatment were pooled into sterile 250 mL beakers to minimize within-treatment variability and to obtain representative composite samples. From each pooled sample, 45 mL aliquots were transferred into sterile tubes and immediately stored at −80 °C for the duration of the 6-day sampling period until further analysis. Pooled samples collected across six consecutive days for each treatment group were subsequently analyzed using liquid chromatography–mass spectrometry (LC–MS). Samples were thawed on ice for approximately 2 h prior to metabolomic profiling. Untargeted metabolite profiling was conducted using chemical isotope labeling (CIL) coupled with LC–MS. Dansylation with ^12^C/^13^C-isotopes was used to enable chemical group-specific detection of metabolites, such as amines/phenols, carboxylic acids, carbonyl compounds (aldehydes and ketones), and hydroxyl-containing metabolites. This method increases metabolite coverage and improves quantification accuracy. Detailed descriptions of the CIL–LC–MS workflow, including labeling procedures and sample preparation, have been published previously [30]. Chromatographic separation was performed on an Agilent Eclipse Plus C18 column (150 × 2.1 mm, 1.8 μm) at 40 °C using a mobile-phase system composed of water with 0.1% formic acid (A) and acetonitrile with 0.1% formic acid (B). A standard CIL gradient elution program was applied following established protocols, and all samples were analyzed in positive electrospray ionization (ESI+) mode on an Agilent 1100 LC system coupled to a Bruker Impact HD QTOF mass spectrometer (Bruker Corporation, Billerica, MA, USA).

In total, 30 raw LC–MS datasets were obtained, including 24 experimental samples (6 replicates per treatment) and 6 pooled quality control (QC) samples. Pooled quality control (QC) samples were prepared by combining equal aliquots of all individual study samples to represent the overall metabolite composition. For LC–MS analysis, 45 μL of each sample, including QCs, was mixed with 5 μL of an internal standard solution to monitor instrument performance and data normalization. QC samples were injected at regular intervals throughout the analytical sequence (every 10 samples) to assess instrument stability and reproducibility. Metabolites exhibiting a relative standard deviation (RSD) ≥ 25% across QC samples were excluded from further analysis, resulting in a median QC coefficient of variation (CV) of 11.8% among retained features. Data preprocessing was performed using IsoMS Pro software (v1.2.16), which removed redundant peaks derived from adducts, multimers, or dimers. Features absent in ≥25% of samples within a treatment group were excluded. Metabolite annotation was based on accurate mass and retention time matches against curated CIL-linked identity libraries, ensuring reliable metabolite identification and minimizing false positives [30]. Metabolite identification confidence adhered to Metabolomics Standards Initiative (MSI) criteria: Level 1 for compounds matched to authentic standards (mass + retention time), Level 2 for putatively annotated compounds (library mass + retention time match), Level 3 for tentative identifications based on accurate mass and isotopic profile, and Level 4 for unknown features.

### 2.4. Untargeted Metabolome Analysis

The processed metabolomic data were imported into MetaboAnalyst 6.0 (https://www.metaboanalyst.ca/ (accessed on 15 August 2025)) for statistical and pathway analyses. Data integrity checks were carried out prior to normalization, which was performed using median-centering, log transformation, and autoscaling as recommended for large-scale metabolomics datasets [31]. Chemometric evaluation was conducted using Partial Least Squares Discriminant Analysis (PLS-DA) to visualize separation patterns between dietary treatments and the control. Model reliability was tested via permutation analysis, assessing R^2^Y (goodness of fit) and Q^2^ (predictive ability). Differentially abundant metabolites were determined using volcano plot analysis, with thresholds set at *p* ≤ 0.05 and fold-change ≥ 1.5. The Benjamini–Hochberg false discovery rate (FDR) procedure was applied to correct for multiple comparisons, and adjusted *q*-values were reported. The partial least squares–discriminant analysis (PLS-DA) model was validated using 5-fold cross-validation with four latent components. Model performance was assessed based on accuracy, R^2^, and Q^2^ values to evaluate stability and predictive reliability.

Receiver Operating Characteristic (ROC) curve analysis was then applied to identify metabolites with strong discriminatory potential. Biomarker candidates were classified based on their area under the curve (AUC) values, where AUCs approaching 1.0 denoted high predictive accuracy [32]. Finally, pathway enrichment analysis was carried out using the Kyoto Encyclopedia of Genes and Genomes (KEGG) database. Pathway enrichment analysis was performed to identify significantly affected metabolic pathways (*p* ≤ 0.05), providing mechanistic insights into the metabolic responses to GEO, MEO, and OLEO treatments relative to the control. For each pathway, the number of matched metabolites (Hits), total pathway size, enrichment ratio (Hits/Total), raw *p*-value, and FDR-adjusted *q*-value were documented.

### 2.5. DNA Extraction of Ruminal Microorganisms

DNA was extracted using the ZymoBIOMICS™ DNA Miniprep Kit (Zymo Research, Irvine, CA, USA) following the manufacturer’s protocol. Briefly, sample pellets were resuspended in 750 μL lysis solution, transferred into ZR BashingBead™ Lysis Tubes, and subjected to mechanical disruption with an MP FastPrep-24™ lysis system (five cycles of 1 min lysis at maximum speed followed by 5 min rest). After centrifugation at 10,000× *g* for 1 min, 400 μL of the supernatant was filtered through a Zymo-Spin™ III-F column. The filtrate was combined with DNA binding buffer, loaded onto a Zymo-Spin™ IICR column, and washed sequentially with ZymoBIOMICS™ DNA Wash Buffers 1 and 2. DNA was eluted in 75 μL of DNase/RNase-free water, purified using a Zymo-Spin™ III-HRC filter, and quantified with the Qubit™ 1X dsDNA assay kit (Thermo Fisher Scientific, Waltham, MA, USA). DNA integrity was further verified by agarose gel electrophoresis to ensure suitability for downstream sequencing.

### 2.6. Sequencing of the 16S rRNA Gene and Diversity Analysis

Microbial community profiling was conducted by targeting the V3–V4 hypervariable regions of the 16S rRNA gene using the Quick-16S™ Next-Generation Sequencing (NGS) Library Prep Kit (Zymo Research, Irvine, CA, USA). Amplification was performed with barcoded, phased fusion primers: 341F (5′-CCTACGGGDGGCWGCAG-3′) and 806R (5′-GACTACNVGGGTMTCTAATCC-3′). Following library cleanup and normalization, pooled samples were sequenced on the Illumina NextSeq 2000 system (Illumina Inc., San Diego, CA, USA) using a P1 600-cycle flow cell to generate paired-end reads (2 × 301 bp). Base calling, demultiplexing, and quality trimming were performed using bcl-convert software (v4.2.4, Illumina Inc., San Diego, CA, USA). Sequence data were processed using the Quantitative Insights Into Microbial Ecology 2 (QIIME 2, version 2022.2) pipeline [33]. Taxonomic classification of amplicon sequence variants (ASVs) was carried out using a Naive Bayes classifier trained on the SILVA rRNA database (version 138), applying a 99% sequence similarity threshold. Representative sequences were aligned against the SILVA reference for taxonomic assignment. Microbial diversity and compositional analyses were performed using MicrobiomeAnalyst 2.0 (https://www.microbiomeanalyst.ca/ (accessed on 19 August 2025)) [34]. Prior to analysis, raw count data were subjected to quality filtering by applying a low-count filter (minimum prevalence threshold: 20%) and removing low-variance features within the 10th percentile based on interquartile range. The resulting data were normalized by rarefaction, followed by total sum scaling (TSS) and relative log expression (RLE) transformation to minimize library size and compositional biases.

Alpha diversity was calculated using the Shannon index, and beta diversity was assessed using weighted and unweighted UniFrac distance matrices. Differential abundance testing was performed within MicrobiomeAnalyst using the normalized feature table, and microbial-metadata associations were evaluated through MaAsLin2 (v1.8.0) employing a multivariable linear model (LM) framework with Benjamini–Hochberg false discovery rate (FDR) correction (adjusted *p* < 0.05). For machine-learning based classification, Random Forest (RF) analysis was performed in MicrobiomeAnalyst under default hyperparameter settings: number of trees = 500, number of predictors per split = 7, and randomization enabled. Model performance was validated using 10-fold cross-validation, and taxa importance was ranked based on mean decrease in accuracy (MDA) scores. All statistical analyses and visualizations were conducted using the integrated R environment (R v4.3.2) within the MicrobiomeAnalyst platform.

Integrative analyses were performed using MicrobiomeAnalyst 2.0. Metabolomic data were mapped to KEGG pathways after prevalence (20%) and variance (10%) filtering, rarefaction, total sum scaling, and relative log expression (RLE) transformation. Pathway enrichment was conducted using Fisher’s exact test with FDR < 0.05. Microbiome–metabolome associations were evaluated in the Multi-Omics Integration module using Spearman correlation (|r| ≥ 0.3, FDR < 0.05). Correlation matrices and network edge tables were generated in MicrobiomeAnalyst and exported to Cytoscape (v3.10.0) for visualization.

## 3. Results

**Figure 1:**
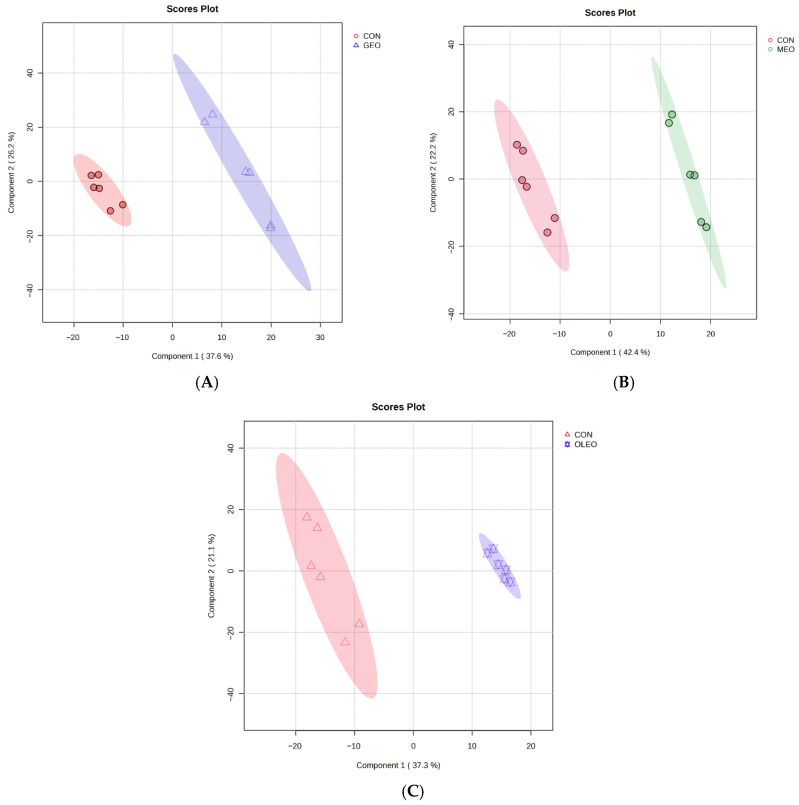
Partial Least Squares Discriminant Analysis (PLS-DA) Scores Plot showing the metabolome between (**A**) CON and GEO group; (**B**) CON and MEO group; and (**C**) CON and OLEO group.

The box plots illustrating metabolomic distributions before and after normalization are presented in Figures S1–S3. A total of 661 unique metabolites were detected and identified (Supplementary Files S1). Fold-change analysis identified 51 significantly upregulated (*p* < 0.05) metabolites and 61 significantly downregulated metabolites in the GEO group (Figure S4). For the MEO treatment, 55 metabolites were significantly upregulated and 78 were downregulated relative to control (Figure S5). In the OLEO group, 20 metabolites were significantly upregulated and 63 downregulated compared with the control (Figure S6). The key differentially abundant metabolites that were significantly influenced are shown in Table 1. Partial Least Squares Discriminant Analysis (PLS-DA) scores plots demonstrated clear separation between treatments and the control, with the first two components explaining 37.6% and 26.2% of the variation for GEO (Figure 1A), 42.4% and 22.2% for MEO (Figure 1B), and 37.3% and 21.1% for OLEO (Figure 1C). These results indicate that supplementation with feed additives significantly reshaped the rumen fluid metabolome. Permutation testing confirmed model robustness, with significant empirical *p*-values for both Q^2^ (*p* ≤ 0.01) and R^2^Y (*p* ≤ 0.01; Figures S7–S9). Together, these findings indicate that dietary treatments induced consistent and significant alterations in the ruminal metabolome, with Variable Importance in Projection (VIP) analysis identifying the key discriminatory features.

**Figure 2:**
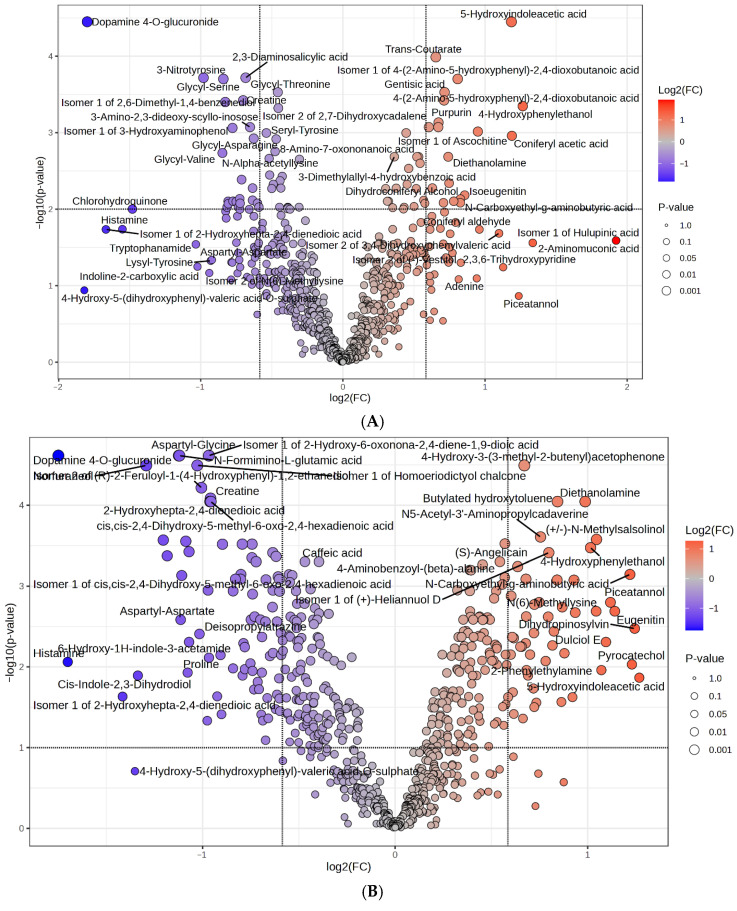

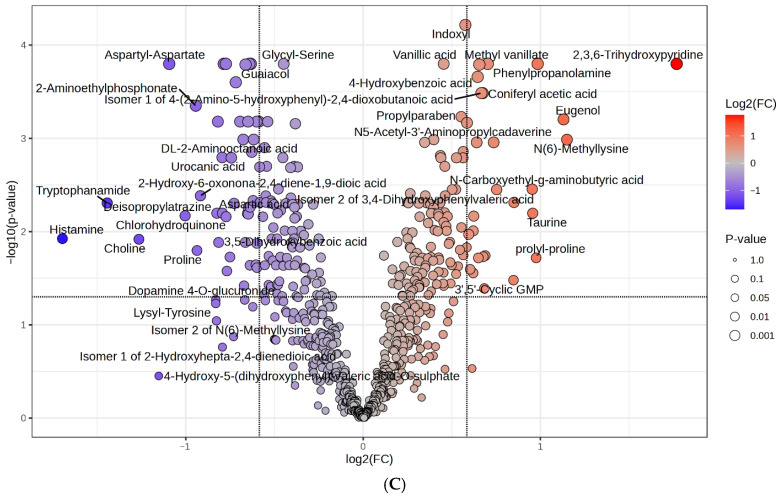
Volcano plot showing the differential rumen metabolites between (**A**) CON and GEO group; (**B**) CON and MEO group; and (**C**) CON and OLEO group.

Volcano plot analysis further highlighted differentially abundant metabolites (fold change ≥ 1.5; *p* ≤ 0.05). In GEO (Figure 2A), 86 metabolites were significantly altered. Notably increased metabolites included Isomer 1 of hulupinic acid (3.79-fold), 2-aminomuconic acid (2.52-fold), 4-hydroxyphenylethanol (2.40-fold), coniferyl acetic acid (2.28-fold), 5-hydroxyindoleacetic acid (2.27-fold), and Isomer 2 of 3,4-dihydroxyphenylvaleric acid (2.14-fold). In contrast, dopamine 4-O-glucuronide (0.29-fold), Isomer 1 of 2-hydroxyhepta-2,4-dienedioic acid (0.31-fold), histamine (0.34-fold), chlorohydroquinone (0.36-fold), and tryptophanamide (0.49-fold) were markedly decreased. In the MEO group (Figure 2B), prominent increases were observed for 5-hydroxyindoleacetic acid (2.41-fold), dihydropinosylvin (2.37-fold), pyrocatechol (2.35-fold), N-carboxyethyl-γ-aminobutyric acid (2.33-fold), eugenitin (2.20-fold), piceatannol (2.17-fold), dulciol E (2.14-fold), and 2-phenylethylamine (2.10-fold). Decreases were detected in dopamine 4-O-glucuronide (0.30-fold), histamine (0.31-fold), Isomer 1 of 2-hydroxyhepta-2,4-dienedioic acid (0.37-fold), cis-indole-2,3-dihydrodiol (0.40-fold), norfuraneol (0.41-fold), 2-hydroxy-6-oxonona-2,4-diene-1,9-dioic acid (0.43-fold), aminoacrylic acid (0.44-fold), and aspartyl-glycine (0.46-fold). In OLEO (Figure 2C), significantly upregulated metabolites included 2,3,6-trihydroxypyridine (3.41-fold), N(6)-methyllysine (2.22-fold), eugenol (2.19-fold), phenylpropanolamine (1.98-fold), prolyl-proline (1.97-fold), taurine (1.94-fold), and N-carboxyethyl-γ-aminobutyric acid (1.94-fold). Downregulated metabolites included chlorohydroquinone (0.50-fold), aspartyl-aspartate (0.47-fold), choline (0.42-fold), tryptophanamide (0.37-fold), and histamine (0.31-fold). These metabolite shifts highlight that each additive combination exerts distinct effects on ruminal biochemical pathways, with both common and unique signatures across treatments.

**Figure 3:**
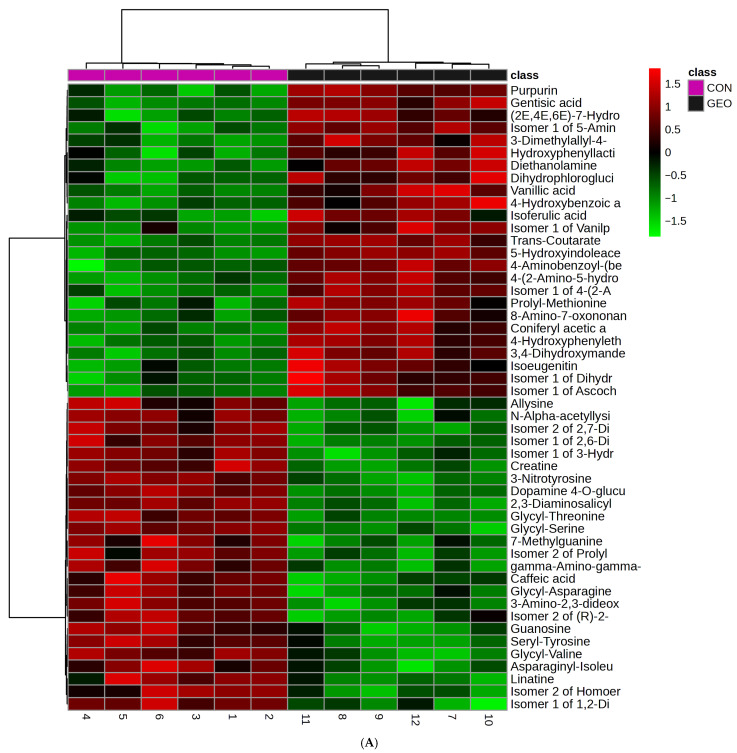

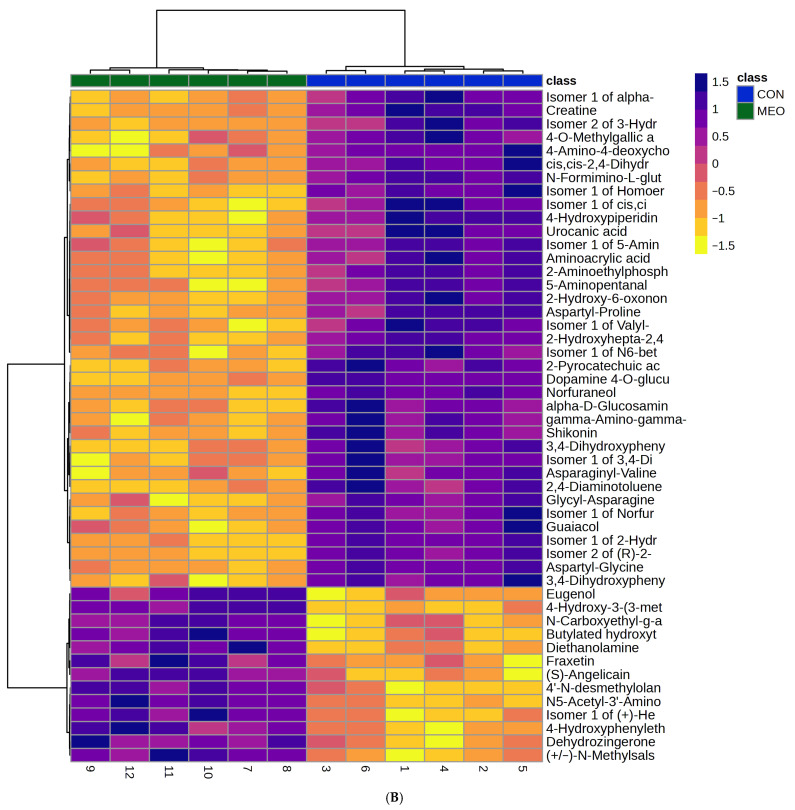

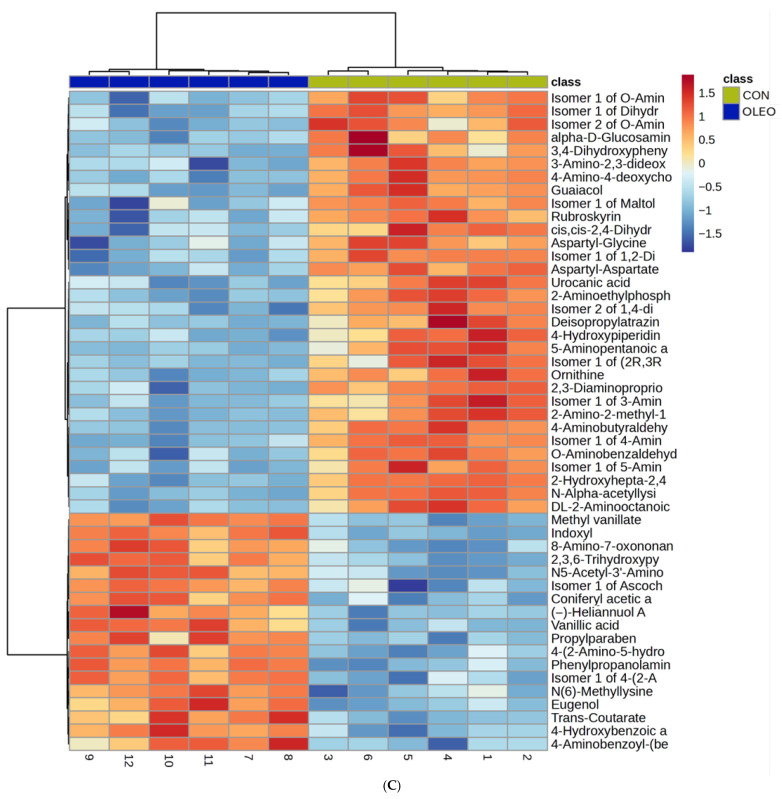
Heatmap showing the 50 top differential rumen metabolites between (**A**) CON and GEO group; (**B**) CON and MEO group; and (**C**) CON and OLEO group.

The VIP scores and heatmap of the top 50 differentially abundant metabolites revealed GEO treatment-specific metabolic profiles (Figure S10 and Figure 3A). GEO supplementation elevated 5-Hydroxyindoleacetic acid, trans-Coutarate, Gentisic acid, 4-Hydroxyphenylethanol, Coniferyl acetic acid, Purpurin, and Ascochitine, whereas the control group showed higher levels of Dopamine 4-O-glucuronide, Glycyl-Serine, 3-Nitrotyrosine, Creatine, and Seryl-Tyrosine. In the MEO group (Figure S11 and Figure 3B), increased metabolites included 4-Hydroxy-3-(3-methyl-2-butenyl)acetophenone, N5-Acetyl-3-aminopropylcadaverine, Butylated hydroxytoluene, Diethanolamine, Eugenol, Fraxetin, and (+/−)-N-Methylsalsolinol, while the control group showed greater abundance of Norfuraneol, Aspartyl-Glycine, Dopamine 4-O-glucuronide, Creatine, Urocanic acid, and Aspartyl-Proline. In the OLEO group (Figure S12 and Figure 3C), elevated metabolites included Indoxyl, Methyl vanillate, Phenylpropanolamine, Vanillic acid, 2,3,6-Trihydroxypyridine, 4-Hydroxybenzoic acid, Coniferyl acetic acid, Eugenol, and N(6)-Methyllysine, whereas the control group exhibited higher concentrations of N-α-acetyllysine, 4-Aminobutyraldehyde, Aspartyl-Aspartate, Guaiacol, 2-Aminoethylphosphonate, and Ornithine. Collectively, these findings indicate that distinct metabolic signatures associated with each feed additive, reflecting their differential impact on ruminal metabolism and microbial activity.

The ruminal metabolites serving as biomarkers were assessed using Receiver–operator characteristic (ROC) curves. According to biomarker utility classification, candidate markers with AUC ≥ 0.9 are considered excellent. In this study, biomarkers with AUC = 1 enhanced by GEO included 4-Hydroxyphenylethanol, 5-Hydroxyindoleacetic acid, Chlorohydroquinone, Coniferyl acetic acid, Dopamine 4-O-glucuronide, Isomer 1 of 2-Hydroxyhepta-2,4-dienedioic acid, and Isomer 1 of Hulupinic acid (Figure S13). MEO-enriched biomarkers included 2,3-Diaminoproprionic acid, 2-Phenylethylamine, 8-Aminooctanoic acid, 2-Pyrocatechuic acid, 5-Aminopentanoic acid, 5-Hydroxyindoleacetic acid, 7-Methylguanine, Alanyl-Alanine, and Aminoadipic acid (Figure S14). OLEO-enhanced biomarkers included Histamine, Tryptophanamide, Choline, 2,3,6-Trihydroxypyridine, N(6)-Methyllysine, Eugenol, Phenylpropanolamine, Isomer 2 of 3,4-Dihydroxyphenylvaleric acid and Taurine (Figure S15).

**Figure 4:**
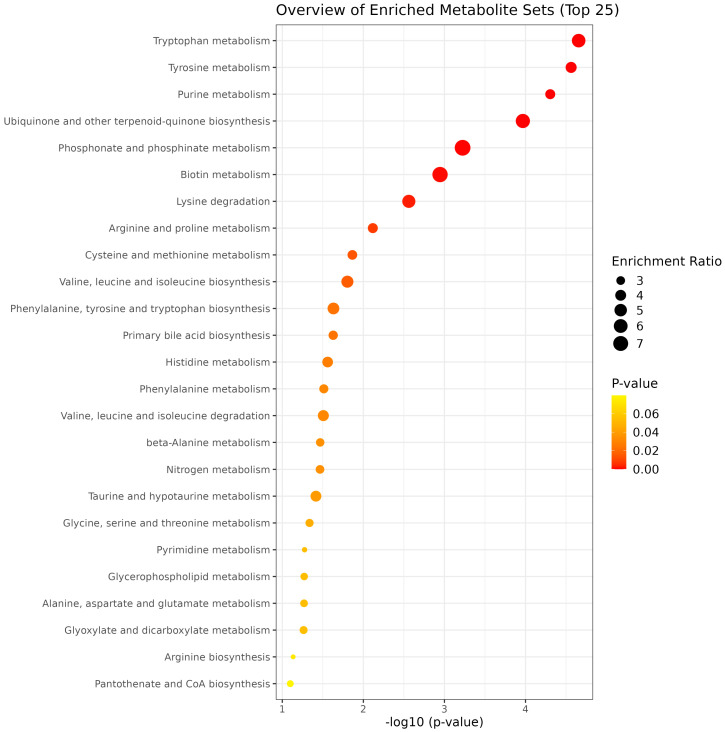
Top 25 metabolic pathways enriched by GEO treatment.

**Figure 5:**
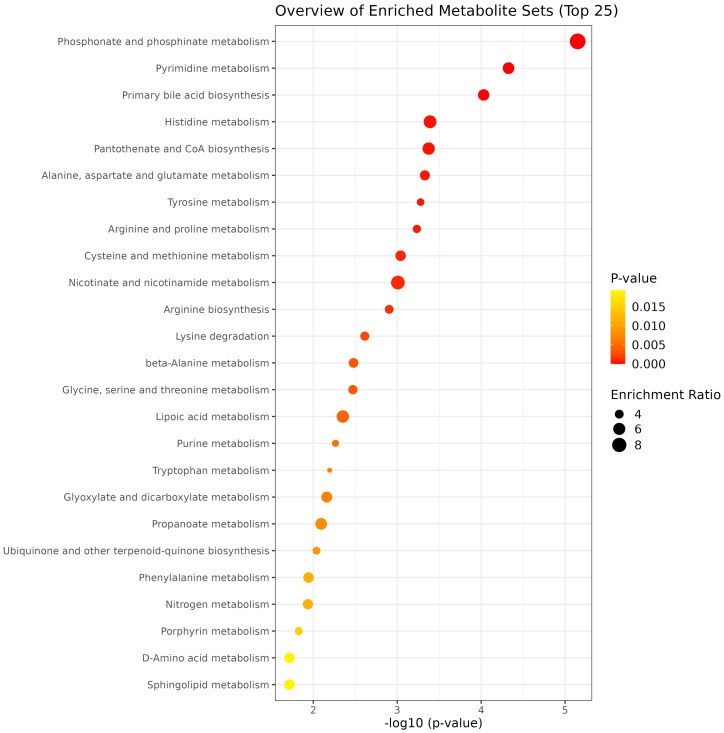
Top 25 metabolic pathways enriched by MEO treatment.

**Figure 6:**
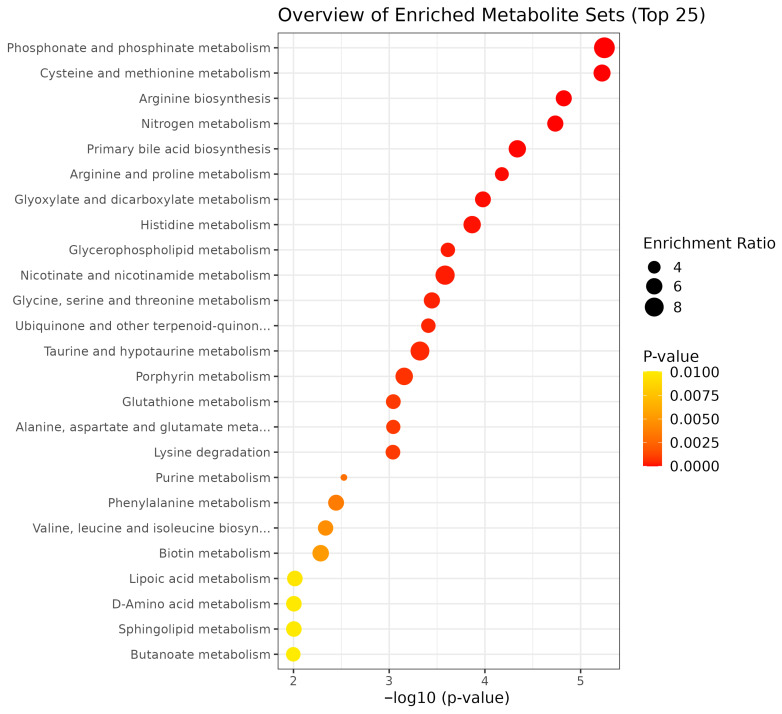
Top 25 metabolic pathways enriched by OLEO treatment.

The enrichment pathway analysis revealed distinct treatment effects. GEO supplementation enriched tryptophan, tyrosine, purine, biotin, phosphonate and phosphinate metabolism, ubiquinone and other terpenoid-quinone biosynthesis, and lysine degradation pathways (Figure 4). MEO supplementation enriched phosphonate and phosphinate, pyrimidine, histidine, alanine, aspartate, and glutamate metabolism, as well as primary bile acid and pantothenate and CoA biosynthesis (Figure 5). OLEO supplementation enriched phosphonate and phosphinate, cysteine and methionine, nitrogen, histidine, nicotinate and nicotinamide, taurine and hypotaurine, porphyrin and glutathione metabolism, as well as arginine and primary bile acid biosynthesis (Figure 6). These enriched pathways suggest that while all treatments influenced amino acid and energy metabolism, OLEO also modulated vitamin-related and bile acid pathways, potentially linking to host-microbial interactions.

**Figure 7:**
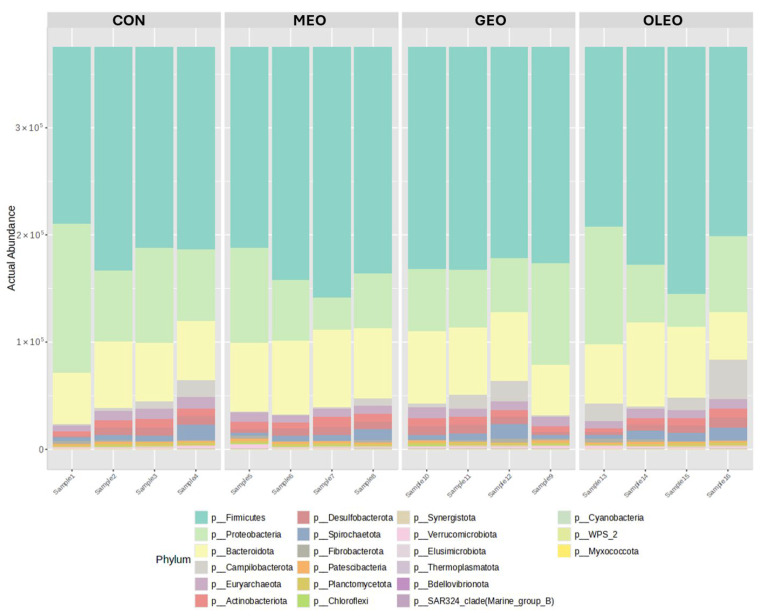
Effect of combined prebiotics (GOS, MOS), EOB and OPE on ruminal heatmap of phylum abundance.

The microbiome results showed no significant differences in overall bacteria, archaea or unassigned counts among treatments compared to the control (Table 2), likely due to higher within-group variability relative to between-group differences. Bacterial abundance ranged from 471,850 in OLEO to 528,844 in CON, while archaea ranged from 9589 in OLEO to 12,124 in CON. At the phylum level (Figure 7; Table 3), a total of 25 different phyla were identified. No significant differences (*p* > 0.05) were observed in the major bacterial phylum including *Firmicutes*, *Proteobacteria*, *Bacteroidota*, *Actinobacteriota*, *Campilobacterota*, *Desulfobacterota*, *Spirochaetota*, *Fibrobacterota*, *Synergistota*, and *Chloroflexi*. Similarly, archaeal phyla *Euryarchaeota* and *Thermoplasmatota* were unaffected by the additives. However, the effects of each additive on the abundance of microbial taxa at the phylum level are shown in Figure S17. Despite the absence of phylum-level changes, genus-level analysis revealed treatment-specific effects.

**Figure 8:**
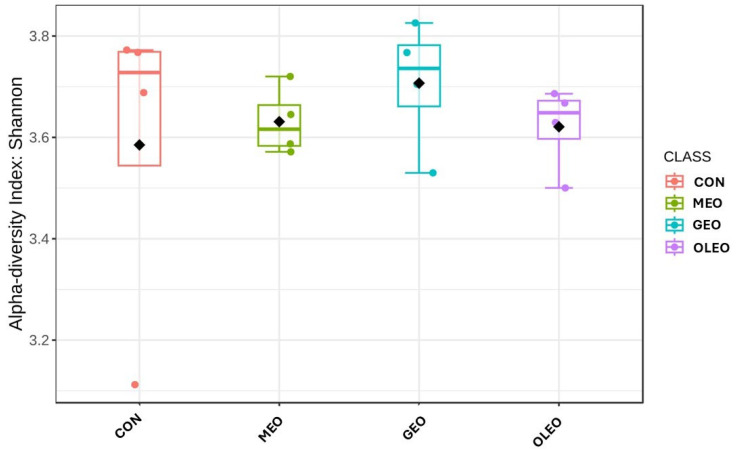
Effect of combined prebiotics (GOS, MOS), EOB and OPE on ruminal alpha diversity Shannon index.

**Figure 9:**
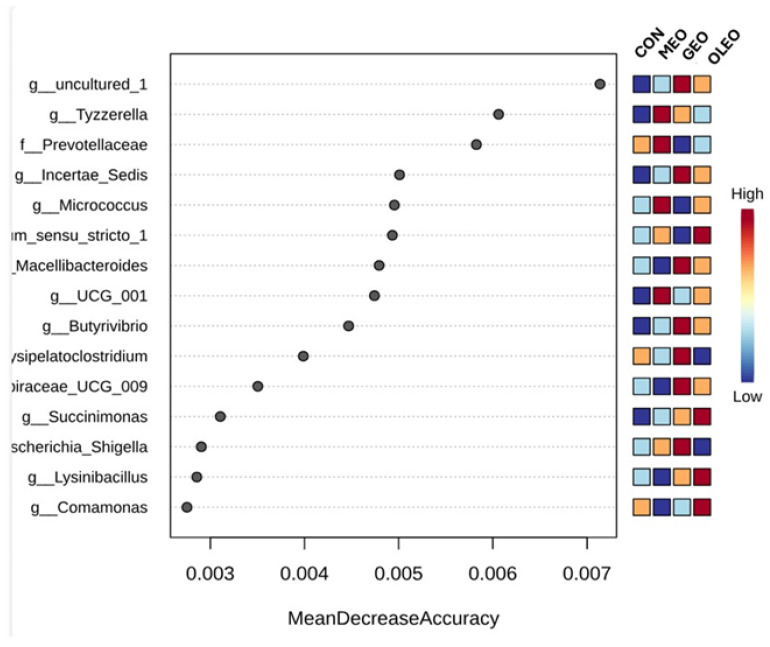
Effect of combined prebiotics (GOS, MOS), EOB and OPE on ruminal genera Random Forest Classification.

The alpha diversity indices at the phylum level are shown in Figure 8. The Random Forest classification analysis at the genus level is shown in Figure 9. The feature importance ranking indicated that Uncultured taxa exhibited the highest mean decrease accuracy (>0.007). Two genera, *Tyzzerella* and *Prevotellaceae*, showed mean decrease accuracy values of 0.006, while *Incertae_sedis*, *Micrococcus*, and *Clostridium_sensu_stricto_1* followed closely with values of 0.005. In addition, *Butyrivibrio*, *Succinimonas*, *Escherichia–Shigella*, *Lysinibacillus*, and *Comamonas* demonstrated mean decrease accuracy scores ranging between 0.003 and 0.005.

Differential abundance analysis was carried out between the control and each dietary treatment using a threshold of Log2 fold-change > 1 and *p* ≤ 0.05. As shown in Table 4, the MEO treatment significantly altered a total of 21 taxa (13 enriched and 8 suppressed). Notable increases included *Pseudarcobacter* (3.49-fold), *Lachnospira* (2.68-fold), *Tyzzerella* (2.58-fold), and *Succinivibrionaceae* (1.89-fold). However, taxa such as *Acinetobacter* (2.84-fold), *Rhodobacteraceae* (2.61-fold), and *Enterobacter* (2.21-fold) were markedly decreased. These shifts suggest that MEO supplementation may promote succinate- and butyrate-producing bacteria while inhibiting opportunistic or potentially pathogenic genera, potentially supporting a more efficient fermentation environment.

Table 5 shows that GEO treatment significantly affected 19 taxa (12 enriched and 7 suppressed). Marked increases were observed for *Uncultured_1* (10.9-fold), *Incertae_Sedis* (6.20-fold), *Macellibacteroides* (5.75-fold), *Erysipelatoclostridium* (4.55-fold), *Lachnospiraceae*_UCG_009 (3.05-fold), *WPS*_2 (2.40-fold), *Escherichia_Shigella* (2.39-fold), and *Tyzzerella* (2.16-fold). Conversely, genera such as *Enterococcus* (2.79-fold), *Tuzzerella* (2.30-fold), *Agathobacter* (2.27-fold) and *Micrococcus* (2.15-fold) were suppressed. These results indicate that GEO promoted the proliferation of underexplored and fiber-degrading taxa while suppressing lactic acid bacteria and opportunistic commensals, pointing to a restructuring of microbial networks toward novel fermentation niches.

As outlined in Table 6, the OLEO treatment altered 22 taxa (13 enriched and 9 suppressed). Significant increases were recorded for *Uncultured_1* (6.76-fold), *Incertae_Sedis* (5.29-fold), *Lysinibacillus* (4.59-fold), *Christensenellaceae* (3.65-fold), *Lachnospira* (3.45-fold), and *Lachnospiraceae*_UCG_009 (2.00-fold). On the other hand, *Enterococcus* (2.83-fold), *Vagococcus* (2.65-fold), *Agathobacter* (2.13-fold), *Catenibacterium* (2.13-fold) and *Enterobacter* (2.03-fold) were strongly suppressed. Together, these findings suggest that OLEO encouraged the enrichment of rare taxa with potential metabolic versatility, while reducing the abundance of lactic acid–producing and opportunistic bacteria, which may contribute to a more stable and diverse microbial ecosystem.

**Figure 10:**
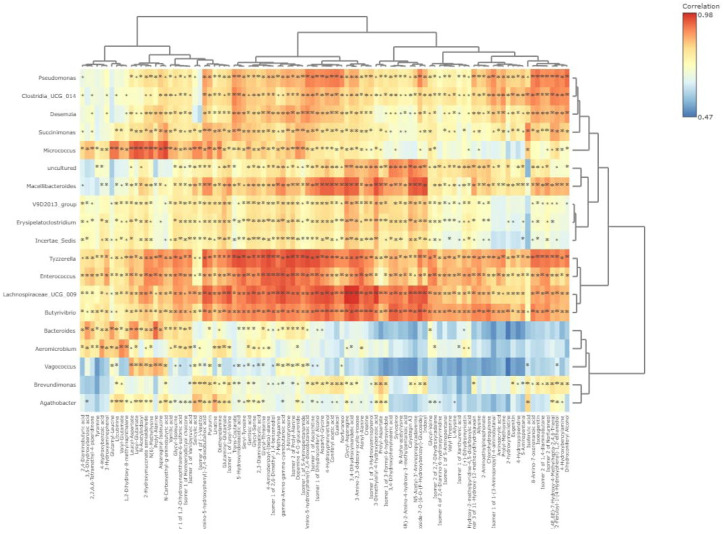
Integrative microbiome-metabolome correlation as influenced by GEO (* Correlation is significant at *p* < 0.05 level).

The microbiome-metabolome correlation heatmap as influenced by GEO supplementation is shown in Figure 10. There was a strong linear correlation (*p* < 0.01) between *Butyrivibrio* and 3,4-Dihydroxymandelic acid (r = 0.95), Cyanidin 3-O-Glucoside-7-O-(6-O-(P-Hydroxybenzoyl)-Glucoside) (r = 0.95), 4-Hydroxyphenylethanol (r = 0.94), Sylvopinol (r = 0.94), 3-Amino-2,3-dideoxy-scyllo-inosose (r = 0.94), Isomer 1 of 3-Hydroxyaminophenol (r = 0.94), Glycyl-Asparagine (r = 0.93), Creatine (r = 0.93) and Isomer 1 of Dihydroconiferyl Alcohol (r = 0.93). *Enterococcus* was linearly (*p* < 0.01) correlated with Isomer 2 of Prolyl-Alanine (r = 0.95), gamma-Amino-gamma-cyanobutanoic acid (r = 0.95), 4-Aminobenzoyl-(beta)-alanine (r = 0.94), Glycyl-Threonine (r = 0.94), 7-Methylguanine (r = 0.94), Isomer 1 of 2,6-Dimethyl-1,4-benzenediol (r = 0.93), 3-Nitrotyrosine (r = 0.93), Alanyl-Alanine (r = 0.93) and Seryl-Tyrosine (r = 0.93). *Lachnospiraceae*_UCG_009 exhibited a strong linear (*p* < 0.01) relationship with Glycyl-Asparagine (r = 0.98), 3,4-Dihydroxymandelic acid (r = 0.98), 3-Amino-2,3-dideoxy-scyllo-inosose (r = 0.98), gamma-Amino-gamma-cyanobutanoic acid (r = 0.96), Isomer 1 of Lysyl-Valine (r = 0.95), Isomer 1 of Dihydroconiferyl Alcohol (r = 0.95), Calystegin A3 (r = 0.95), Coniferyl acetic acid (r = 0.95), Creatine (r = 0.94), Cyanidin 3-O-Glucoside-7-O-(6-O-(P-Hydroxybenzoyl)-Glucoside) (r = 0.94), Isomer 1 of Ascochitine (r = 0.95) and Purpurin (r = 0.94).

A strong correlation (*p* < 0.01) existed between *Macellibacteroides* and Indoxyl (r = 0.95), Glycyl-Asparagine (r = 0.95), Cyanidin 3-O-Glucoside-7-O-(6-O-(P-Hydroxybenzoyl)-Glucoside) (r = 0.94), Calystegin A3 (r = 0.94), Coniferyl acetic acid (r = 0.94), Aspartyl-Glycine (r = 0.94), 3-Amino-2,3-dideoxy-scyllo-inosose (r = 0.93), 4-Hydroxyphenylethanol (r = 0.93), and 3,4-Dihydroxymandelic acid (r = 0.93). *Micrococcus* was strongly (*p* < 0.01) correlated with N-Carboxyethyl-g-aminobutyric acid (r = 0.95), Lysyl-Glutamate (r = 0.95), Asparaginyl-Isoleucine (r = 0.94), N(6)-Methyllysine(r = 0.94), and Leucyl-Aspartate (r = 0.93). *Tyzzerella* also demonstrated a strong correlation (*p* < 0.01) with 4-(2-Amino-5-hydroxyphenyl)-2,4-dioxobutanoic acid (r = 0.96), Isomer 2 of Prolyl-Alanine (r = 0.96), 3-Nitrotyrosine (r = 0.96), 4-Aminobenzoyl-(beta)-alanine (r = 0.96), Isomer 1 of Dihydroconiferyl Alcohol (r = 0.95), Seryl-Tyrosine (r = 0.95), Trans-Coutarate (r = 0.95), Isomer 1 of 5-Aminopentanamide (r = 0.95), 4-Hydroxyphenylethanol (r = 0.93), Aspartyl-Glycine (r = 0.93), and Isoeugenitin (r = 0.93).

**Figure 11:**
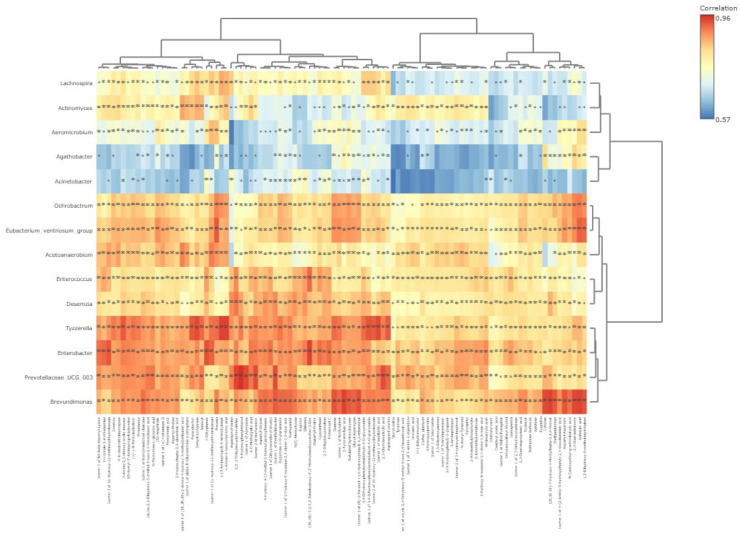
Integrative microbiome-metabolome correlation as influenced by MEO (* Correlation is significant at *p* < 0.05 level).

The integrative microbiome-metabolome correlation as influenced by MEO supplementation (Figure 11) indicated a strong linear correlation (*p* < 0.01) between *Brevundimonas* and Isoferulic acid (r = 0.96), 2-Pyrocatechuic acid (r = 0.96), 2,4-Diaminotoluene (r = 0.95), Cytidine (r = 0.95), Isomer 2 of (R)-2-Feruloyl-1-(4-Hydroxyphenyl)-1,2-ethanediol (r = 0.95), Guanine (r = 0.94), 1,2-Dihydroxy-8-methylnaphthalene (r = 0.94), Aspartyl-Aspartate (r = 0.94), Diethanolamine (r = 0.94), Butylated hydroxytoluene (r = 0.94), Isomer 1 of Deethylatrazine (r = 0.94), Glycyl-Asparagine (r = 0.93), Dopamine 4-O-glucuronide (r = 0.93), Isomer 1 of 2-Hydroxy-6-oxonona-2,4-diene-1,9-dioic acid (r = 0.93), Isomer 1 of Norfuraneol (r = 0.93), and Norfuraneol (r = 0.93).

A strong correlation (*p* < 0.01) existed between *Prevotellaceae_UCG_003* and 4-Hydroxyphenylethanol (r = 0.97), 2,2′,3-Trihydroxydiphenylether (r = 0.95), Isomer 1 of Adenosine (r = 0.95), Isomer 1 of Deethylatrazine (r = 0.95), Isomer 2 of Norfuraneol (r = 0.95), 2,3-Diaminosalicylic acid (r = 0.95), and Asparaginyl-Leucine (r = 0.93). *Tyzzerella* was strongly (*p* < 0.01) correlated with 4-Amino-4-deoxychorismic acid (r = 0.96), 1-(3-Aminopropyl)-4-aminobutanal (r = 0.96), Isomer 1 of Vanillactic acid (r = 0.95), Dehydrozingerone (r = 0.95), Isomer 1 of 3,4-Dihydroxyphenylacetaldehyde 4-O-glucuronide (r = 0.94), Pyrocatechol (r = 0.94), 3-Amino-2,3-dideoxy-scyllo-inosose (r = 0.94), Asparaginyl-Leucine (r = 0.94), Isomer 2 of 11-Hydroxy-12-methoxydihydrokawain (r = 0.94), Indoxyl (r = 0.93), and Guanine (r = 0.93).

*Ochrobactrum* exhibited a strong linear (*p* < 0.01) relationship with Isoferulic acid (r = 0.91), Fraxetin (r = 0.91), 2,4-Diaminotoluene (r = 0.91), 1,2-Dihydroxy-8-methylnaphthalene (r = 0.90), and 1-(3-Aminopropyl)-4-aminobutanal (r = 0.90). *Eubacterium_ventriosum_group* was linearly (*p* < 0.01) correlated with Fraxetin (r = 0.94), Isoferulic acid (r = 0.93), 1,2-Dihydroxy-8-methylnaphthalene (r = 0.93), 2,4-Diaminotoluene (r = 0.90), (S)-Angelicain (r = 0.90), and Guanine (r = 0.90). *Enterobacter* had a positive correlation with 4-Ethylphenol (r = 0.95), Isomer 3 of 11-Hydroxy-12-methoxydihydrokawain (r = 0.94), 1H-Indole-3-methanamine (r = 0.93), and Heliannuol C (r = 0.93) while *Desemzia* was linearly (*p* < 0.01) correlated with 2,2′,3-Trihydroxydiphenylether (r = 0.92), Isomer 1 of Deethylatrazine (r = 0.90), Asparaginyl-Valine (r = 0.90), N(6)-Methyllysine (r = 0.90), and Shikonin (r = 0.90).

**Figure 12:**
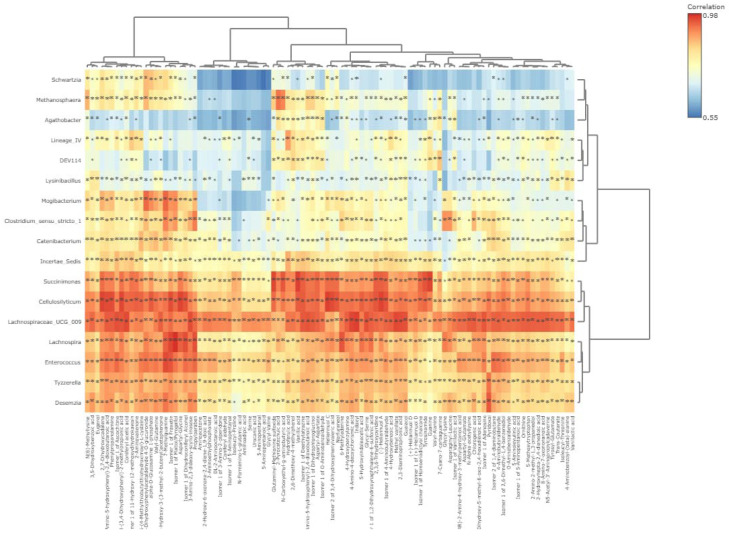
Integrative microbiome-metabolome correlation as influenced by OLEO (* Correlation is significant at *p* < 0.05 level).

The integrative microbiome-metabolome correlation as influenced by OLEO supplementation (Figure 12) revealed a strong linear correlation (*p* < 0.01) between *Cellulosilyticum* and Heliannuol C (r = 0.97), 2,7-Dihydroxycadalene (r = 0.97), Aspartyl-Glycine (r = 0.97), 3-(3,4-Dihydroxyphenyl)-2-methylpropionic acid (r = 0.96), 4-(2-Amino-5-hydroxyphenyl)-2,4-dioxobutanoic acid (r = 0.96), Isomer 2 of 3,4-Dihydroxyphenylvaleric acid (r = 0.96), Isomer 1 of Fraxetin (r = 0.96), Isomer 1 of 4-Aminobutyraldehyde (r = 0.95), 2,3,6-Trihydroxypyridine (r = 0.95), Norfuraneol (r = 0.95), alpha-D-Glucosamine 1-phosphate (r = 0.95), Isomer 1 of Deethylatrazine (r = 0.95), 2-Aminovalienone (r = 0.94) and 5-Hydroxyindoleacetic acid (r = 0.94). Notably, *Lachnospiraceae_UCG_009* was strongly (*p* < 0.01) correlated with Methyl vanillate (r = 0.96), 3-Amino-2,3-dideoxy-scyllo-inosose (r = 0.96), 2,3-Diaminoproprionic acid (r = 0.96), Isomer 1 of Ascochitine (r = 0.96), Guaiacol (r = 0.96), Isomer 1 of Adenosine (r = 0.95), Chlorogenic acid (r = 0.95), 4-Hydroxy-3-(3-methyl-2-butenyl)acetophenone (r = 0.95), Isomer 1 of 1,2-Dihydroxynaphthalene-6-sulfonic acid (r = 0.95), N5-Acetyl-3′-Aminopropylcadaverine (r = 0.94), 4-Hydroxybenzoic acid (r = 0.94), Isomer 1 of 4-Aminobutyraldehyde (r = 0.94) and 3,5-Dihydroxybenzoic acid (r = 0.94).

A strong correlation (*p* < 0.01) existed between *Succinimonas* and Vanillic acid (r = 0.97), Guanine (r = 0.97), Tyrosinamide (r = 0.96), N-Carboxyethyl-g-aminobutyric acid (r = 0.95), 2,3,6-Trihydroxypyridine (r = 0.95), (−)-Heliannuol A (r = 0.95), Isomer 1 of 4-Aminobutyraldehyde (r = 0.94), Aspartyl-Aspartate (r = 0.94), Isomer 1 of Homoeriodictyol chalcone (r = 0.94), Heliannuol C (r = 0.94), Isomer 2 of 3,4-Dihydroxyphenylvaleric acid (r = 0.94), and Norfuraneol (r = 0.94). *Lachnospira* had a positive correlation (*p* < 0.01) with Isomer 1 of Maltol/Pyrogallol (r = 0.98), Isomer 1 of Fraxetin (r = 0.96), Isomer 1 of Dihydroconiferyl Alcohol (r = 0.96), Propylparaben (r = 0.95), 6-Methyladenine (r = 0.95), Aspartyl-Glycine (r = 0.95), 7-Methylguanine (r = 0.95), 5-Hydroxyindoleacetic acid (r = 0.93), and 3-Amino-2,3-dideoxy-scyllo-inosose (r = 0.93).

*Enterococcus* was strongly (*p* < 0.01) correlated with 7-Methylguanine (r = 0.96), Isomer 1 of Fraxetin (r = 0.95), Propylparaben (r = 0.94), Isomer 1 of Ascochitine (r = 0.94), 4-Aminobutyraldehyde (r = 0.93), 4-(2-Amino-5-hydroxyphenyl)-2,4-dioxobutanoic acid (r = 0.93), Valyl-Glutamine (r = 0.93), and 3-Amino-2,3-dideoxy-scyllo-inosose (r = 0.93). *Mogibacterium* was strongly (*p* < 0.01) correlated with 7-Methylguanine (r = 0.94), 3,4-Dihydroxyphenylacetaldehyde 4-O-glucuronide (r = 0.94) and alpha-D-Glucosamine 1-phosphate (r = 0.91). *Desemzia* exhibited a strong linear (*p* < 0.01) relationship with Pyridoxal (r = 0.95), 7-Methylguanine (r = 0.93), Isomer 1 of Fraxetin (r = 0.91), and Propylparaben (r = 0.91).

## 4. Discussion

The metabolomic shifts induced by GEO, MEO, and OLEO reflect coordinated changes in host–microbe co-metabolism, phenolic biotransformation, and nitrogen/amine turnover in the rumen. Both GEO and MEO increased 5-hydroxyindoleacetic acid (5-HIAA), and an important metabolite derived from tryptophan catabolism. 5-HIAA, the principal oxidative metabolite of serotonin (5-HT), serves as a biomarker of serotonergic turnover and an active mediator of rumen function. In ruminants, elevated 5-HIAA reflects altered host–microbiota tryptophan metabolism and is associated with tissue repair and stress adaptation during non-lactating periods [35,36]. Functionally, 5-HIAA acts through G-protein-coupled receptor 35 (GPR35) to modulate mucosal immunity and epithelial integrity, influencing ruminal absorption and fermentation efficiency [37]. By affecting serotonergic control of motility and secretion, 5-HIAA indirectly alters substrate flow and VFA production [38,39]. Changes in 5-HIAA may influence gut motility, epithelial integrity, and immune signaling [34,38]. Thus, 5-HIAA links microbial metabolism and host immune-metabolic regulation, contributing to rumen homeostasis and animal physiological adaptation. Similarly, 2-aminomuconic acid, an intermediate in the kynurenine pathway, increased with GEO supplementation, suggesting enhanced oxidative catabolism of tryptophan and aromatic compounds. This metabolic redirection can affect host energy availability and microbial hydrogen balance, with potential impacts on VFA production and CH_4_ formation [40]. Increases in indoxyl with OLEO treatment and related indole derivatives indicate microbial metabolism of tryptophan. These compounds act as ligands for the aryl hydrocarbon receptor (AhR), modulating intestinal barrier function and immunity [41]. Given the centrality of indole signaling to gut integrity, these changes could have downstream effects on nutrient absorption and systemic inflammation. In dairy production contexts, such shifts in tryptophan metabolism may also translate into improved feed intake behavior and higher milk protein yield, as suggested by previous studies on rumen-protected tryptophan supplementation.

Multiple plant-derived phenolics and their microbial transformation products were increased across treatments. These include coniferyl acetic acid (GEO), piceatannol (MEO), pyrocatechol (MEO), and eugenol (OLEO). These compounds originate from essential oils, onion peel polyphenols, and feed plant secondary metabolites; rumen microbiota transform parent phenolics into smaller phenolic acids and catechols. They possess antioxidant and antimicrobial properties, modulating rumen microbial composition and reducing oxidative stress [42,43]. Piceatannol, a hydroxylated analog of resveratrol, exhibits strong antioxidant and anti-inflammatory properties by modulating signaling pathways such as JAK1, Syk, and NF-κB [44,45,46]. In dairy cows, these activities may help reduce oxidative stress and inflammatory responses, supporting rumen epithelial integrity and overall immune competence. As a phytoestrogen, piceatannol also interacts with estrogen receptors, which could influence reproductive physiology [47]. Collectively, its bioactivity suggests potential benefits for enhancing health and productivity in high-producing cows. These results further indicate that natural feed additives like EOB and OPE may serve as sustainable alternatives to synthetic antioxidants or growth promoters, offering both health benefits and potential productivity gains.

Elevated phenolic metabolites can therefore reduce oxidative stress in the rumen epithelium and systemic circulation while also selectively suppress methanogenic or proteolytic populations, mechanisms that support improved feed efficiency and lower GHG emissions. For example, eugenol selectively inhibits methanogens and proteolytic bacteria, potentially lowering CH_4_ emissions and improving nitrogen utilization [48]. Phenylethylamine, upregulated in MEO, is a microbial decarboxylation product of phenylalanine that influences host neurotransmission and microbial competition. Elevated levels suggest altered proteolytic activity [49]. Conversely, histamine was consistently downregulated across treatments. High ruminal histamine is associated with subacute ruminal acidosis (SARA), epithelial damage, and systemic inflammation [50]. Reduced histamine therefore indicates a beneficial effect of the additives on rumen health and inflammatory status. Dopamine 4-O-glucuronide was decreased in all additive groups, suggesting altered monoamine metabolism and conjugation. While its precise significance in cattle remains unclear, reductions may reflect lower microbial deconjugation or altered hepatic clearance. From a practical standpoint, the concurrent reduction in histamine and dopamine metabolites indicates that these additives may lower the risk of inflammatory ruminal disorders such as SARA, thereby contributing to more stable rumen fermentation and improved animal welfare.

Both N(6)-methyllysine and taurine levels increased with OLEO supplementation. N(6)-methyllysine levels were elevated, indicating increased protein turnover and methylation processes. Okedoyin et al. [51] found that N(6)-methyllysine, a product of lysine methylation, was significantly higher in an EOB containing anise, clove, oregano, and peppermint, compared to the control group. Lysine methylation plays a crucial role in regulating DNA repair, transcription, and replication by modulating effector molecules. Sidney et al. [52] observed that less-efficiently fed cattle had lower levels of N(6)-methyllysine in their stomach fluid, which was associated with poorer growth performance. Elevated N(6)-methyllysine concentrations may promote tissue protein synthesis, including collagen production, immune antibody formation, enzyme, and hormone synthesis, and may help maintain nitrogen balance. Taurine contributes to antioxidant defense, bile acid conjugation, and osmoregulation in ruminants, with positive effects on mammary and hepatic function [53]. Choline was decreased in OLEO. As a methyl donor, choline supports liver lipid metabolism and milk production, lower concentrations in rumen fluid could reflect increased microbial uptake, potentially requiring supplementation under high-producing conditions [54]. Hulupinic acid (upregulated in GEO) and 2,3,6-trihydroxypyridine (upregulated in OLEO) are plant-derived or microbial oxidation products. Their presence demonstrates ruminal transformation of dietary polyphenols, highlighting metabolomics as a tool for tracking additive bioactivity [55]. Together, these changes suggest that OLEO not only improves redox balance but may also enhance protein utilization efficiency, which is critical for maximizing milk protein synthesis in high-yielding cows.

Chlorohydroquinones are halogenated phenolic transformation products produced during microbial degradation of chlorinated aromatics and are indicative of reductive dehalogenation activity by specialized anaerobic microbes. Their presence in rumen fluid may reflect microbial detoxification of plant phenolics or xenobiotics and could transiently alter redox chemistry and electron flow in the rumen (potentially affecting H_2_ availability and methanogenesis). Chlorinated phenolics can also exert antimicrobial effects at sufficiently high concentrations, so detection at elevated levels could signal selective pressure on certain rumen taxa [56]. Isomers of 2-hydroxyhepta-2,4-dienedioic acid (plant/phenylpropanoid-derived dicarboxylate) are typical intermediates in microbial breakdown of lignin/phenylpropanoid-derived plant polymers. Increased or decreased abundance can indicate shifts in the rumen’s capacity to depolymerize lignin-derived components and thus affect fiber accessibility and fermentative VFA yields [57]. Changes in such dicarboxylates therefore plausibly reflect altered lignin-depolymerizing activity (affecting fiber digestibility and energy availability). This finding is especially relevant to dairy production systems where maximizing fiber digestibility is directly linked to feed efficiency and milk output. Improved lignin breakdown may reduce reliance on high-cost concentrates while maintaining energy supply.

2,3-Diaminopropionic acid (L-Dap) is a non-proteinogenic amino acid used by microbes as a building block for siderophores and some antibiotics (e.g., staphyloferrin B). Its detection in the rumen metabolome likely reflects microbial biosynthesis of iron-binding metabolites (siderophores) under iron-limited conditions [58]. Siderophore production alters iron availability and can remodel microbial competition (favoring microbes that can sequester iron), which in turn influences community composition, fermentation end-products, and microbial protein synthesis [59]. 8-Aminooctanoic acid (8-aminocaprylic acid) has been reported in microbial and mammalian biochemical studies as modulators of aminergic/GABAergic pathways or as intermediates in amino-fatty acid catabolism. In the rumen context, such medium-chain amino-fatty acids may originate from microbial catabolism of dietary lipids or non-canonical amino-acid metabolism; they may interfere with the structural integrity of microbial cell membranes. This can alter membrane permeability, compromise the function of membrane-bound proteins, and potentially inhibit microbial growth or alter population dynamics [60]. The detection of siderophore-related metabolites and unusual amino-fatty acids highlights the ecological complexity of the rumen and suggests that dietary additives may indirectly influence microbial competition, resilience under nutrient limitations, and overall fermentation stability.

5-Aminopentanoic acid (5-aminovaleric acid) is a common product of microbial lysine catabolism. In the rumen, elevated levels point to shifts in amino-acid fermentation: increased catabolism of lysine can release nitrogen as ammonia or be channeled into microbial VFA production (affecting microbial protein synthesis efficiency). Because lysine is an indispensable amino acid for the host, enhanced lysine fermentation (and accumulation of its catabolites such as 5-aminovalerate) may imply reduced true protein supply to the animal and altered N-utilization efficiency [61]. 2-Pyrocatechuic acid, also known as 2,3-dihydroxybenzoic acid (2,3-DHBA), is a benzoic acid derivative that plays a critical role in microbial aromatic-compound metabolism and acts as a chelating agent in bacterial siderophores, which are molecules that bind strongly to iron. In the rumen, 2-pyrocatechuic acid affects iron cycling, regulates the oxidative status of the rumen fluid, and indirectly impacts the efficiency of microbial protein synthesis [62]. Phenylpropanolamine (PPA) is a phenethylamine-type sympathomimetic that shares structural similarities with amphetamine and ephedrine, and could exert vasoactive or adrenergic effects upon absorption, potentially impacting gut motility, blood flow to the udder, or systemic cardiovascular tone [63]. The extent of these effects would depend on the amount of PPA produced, the absorption rate, and the sensitivity of the animal. Because of known pressor effects in humans [64], detection warrants cautious interpretation but could point to plant-derived alkaloid metabolism or microbial biotransformation of tyrosine/phenylalanine derivatives. Collectively, the metabolite changes observed indicate that combinations of EOB, OPE, and prebiotics redirect aromatic amino acid catabolism, enrich phenolic-derived antioxidants, and lower pro-inflammatory amines (e.g., histamine). These biochemical shifts are consistent with improved rumen epithelial protection, altered microbial hydrogen economy (favoring propionate over methanogenesis), and potential improvements in systemic health and milk synthesis efficiency. Importantly, these metabolomic changes provide mechanistic evidence for how natural additives may reduce CH_4_ emissions and nitrogen losses, thereby contributing to climate-smart livestock production strategies.

The metabolomic profiles indicate that each additive modulated distinct pathways: GEO primarily enhanced tryptophan, tyrosine, and purine metabolism; MEO upregulated phosphonate and pyrimidine metabolism, and bile acid biosynthesis; and OLEO strengthened phosphonate, nicotinamide, and taurine metabolism. Such differential pathway activation suggests that GEO favors amino acid and nucleotide turnover, MEO supports microbial growth and lipid assimilation, and OLEO bolsters redox balance and cofactor cycling. In GEO, stimulation of tryptophan and tyrosine metabolism may facilitate production of bioactive molecules such as serotonin and niacin (vitamin B_3_). In dairy cows, enhanced tryptophan flux has been associated with improved feed intake and higher milk protein yield when supplemented as rumen-protected forms [65], likely via improved amino acid supply to the mammary gland. Similarly, enhanced purine metabolism signals increased nucleotide turnover and microbial proliferation, which may support microbial protein synthesis and thus augment host amino acid supply [66]. This suggests that GEO may not only optimize microbial efficiency but also contribute to improved nitrogen utilization and potentially reduce nitrogen excretion, which is important for both animal productivity and environmental sustainability. In this way, GEO’s metabolic shifts align with improved nutrient assimilation and reduced nitrogen losses. MEO’s upregulation of pyrimidine and bile acid pathways suggests augmented microbial replication and lipid digestion. Active pyrimidine synthesis is vital for expansion of microbial populations, while bile acids can facilitate lipid emulsification and absorption, an important consideration in high-fat diets. This may be especially relevant for dairy cows in early lactation, when energy demands are high and lipid mobilization is substantial, as improved lipid digestion could mitigate metabolic disorders such as ketosis. These shifts could help dairy cows more efficiently utilize dietary fats and support milk fat synthesis under nutrient-limited conditions. OLEO’s enhancement of nicotinamide (NAD^+^/NADP^+^ precursor) metabolism and taurine pathways points toward strengthened energy metabolism and antioxidant defense. Nicotinamide is central to cellular redox reactions, and taurine supports bile conjugation, osmoregulation, and antioxidative capacity; supplementation of taurine in bovine mammary cells was shown to influence purine and lipid metabolism [67]. By strengthening these pathways, OLEO may help cows maintain redox homeostasis under metabolic stress. Such effects could translate into improved resilience during periods of oxidative or inflammatory stress (e.g., heat stress or transition period), thereby supporting sustained milk production and animal welfare.

The microbial composition analysis revealed no significant differences in bacterial and archaeal abundance or phylum-level distributions across treatments compared to the control. This finding aligns with Thomas et al. [68] and Ike et al. [69], who suggested that feed additives may not produce significant effects at higher taxonomic levels such as the phylum level. The bacterial abundance ranged from 471,850 to 528,844, while archaea ranged from 9589 to 12,124 across treatments, with substantial within-group variability likely masking potential between-group differences. The dominance of *Firmicutes*, *Proteobacteria*, and *Bacteroidota*, which typically constitute over 90% of ruminant gut microbiota, was observed across all treatments. Firmicutes primarily produce butyric acid to support intestinal mucosal repair and barrier function, while *Bacteroidota* generates acetic and propionic acids that inhibit cholesterol synthesis and prevent metabolic diseases. Despite the known antimicrobial properties of essential oils, particularly against Gram-positive bacteria [70], the EOB inclusion in GEO, MEO, and OLEO treatments did not significantly alter phylum abundances. This stability indicates that the tested additive combinations can modulate functional outputs (metabolites, fermentation) without disrupting the core rumen microbiome structure, which is important for maintaining long-term digestive efficiency. This contrasts with previous studies where essential oil compounds influenced microbial profiles, possibly due to differences in dosage, composition, or experimental conditions.

The lack of significant effects on minor phyla such as *Proteobacteria*, *Actinobacteriota*, *Spirochaetota*, and *Fibrobacterota* suggests that the combination of prebiotics (MOS and GOS) with EOB and OPE maintained microbial stability without causing substantial ecological disruption. This stability is particularly relevant in the context of dysbiosis, which refers to an imbalance in the composition or function of microbial populations and is often characterized by reduced diversity, enrichment of potentially pathogenic taxa, and depletion of beneficial species [17]. Such imbalances can perturb key metabolic pathways, impair fermentation, alter VFA profiles, and promote the accumulation of toxic metabolites [18,19]. In cattle, these disturbances have been associated with metabolic disorders including obesity, insulin resistance, hepatic steatosis, and inflammatory conditions, underscoring the importance of interventions that maintain microbial equilibrium. The observed stability also implies that such feed additive strategies may be safely applied without risking adverse shifts in rumen ecology, a key consideration for on-farm implementation. The rumen microbiome exists in constant competition for environmental conditions and resources, and the balanced formulation of these additives may have prevented competitive advantages that typically shift community structure, maintaining functional equilibrium despite supplementation.

The differential enrichment of microbial taxa at the genus level in response to prebiotics, EOB, and OPE highlights distinct modes of action of these additives in shaping rumen ecology and function. *Prevotella* (*Prevotellaceae*) are among the most abundant rumen taxa, with versatile metabolic capacity to degrade non-cellulosic polysaccharides and proteins, producing succinate and propionate [71]. Their enrichment is often associated with enhanced carbohydrate utilization, improved microbial protein synthesis, and greater propionate supply, which supports glucogenic energy and milk protein/lactose production [72]. *Tyzzerella* is less characterized in ruminants but has been reported in gut microbiome studies as diet-responsive and sometimes linked with host metabolic perturbations. In cattle, its presence may indicate shifts in microbial community structure under specific dietary interventions, although its exact ruminal role remains unclear [73]. *Micrococcus* species are primarily environmental or skin commensals that occasionally appear in rumen and milk microbiomes. While not major fermenters, their presence may reflect hygiene or environmental contamination, and in dairy systems they are occasionally associated with mastitis or spoilage organisms rather than beneficial rumen function [74].

*Clostridium_sensu_stricto_1* includes strictly anaerobic, spore-forming bacteria, some of which contribute to protein and amino acid fermentation in the rumen. They can produce butyrate and other metabolites important for gut health, though overrepresentation may also indicate excessive proteolysis and potential ammonia accumulation [75,76]. This dual role highlights the importance of balancing protein supplementation to avoid excess ruminal nitrogen losses while still supporting fibrolytic and butyrogenic activity. Butyrivibrio species are essential fibrolytic bacteria that degrade hemicellulose and participate in biohydrogenation of dietary unsaturated fatty acids. They produce butyrate, a critical energy source for rumen epithelium and a modulator of gut development and barrier function [77,78]. *Succinimonas*, such as *S. amylolytica*, are succinate-producing amylolytic bacteria. Their activity channels fermentation intermediates toward the succinate–propionate pathway, improving glucogenic energy supply and potentially reducing CH_4_ production if succinate is efficiently converted to propionate [79,80]. Thus, enrichment of *Succinimonas* may represent a dual benefit for productivity (more propionate) and environmental sustainability (less CH_4_). *Comamonas* are versatile *Proteobacteria* capable of degrading aromatic compounds and xenobiotics, suggesting a role in metabolizing plant secondary metabolites like phenolics [81]. Their metabolic traits could enhance ruminal detoxification, resilience to dietary phytochemicals, and introduce new pathways such as denitrification that affect nitrogen cycling. Such traits may be particularly relevant when feeding byproducts (e.g., onion peels, distillers’ grains) rich in secondary metabolites, thereby increasing the practical applicability of additive combinations in sustainable livestock systems. These functions may be leveraged to improve milk production and overall bovine health [66].

Supplementation with MEO promoted the abundance of *Pseudarcobacter* and *Succinivibrionaceae*, both of which have been linked to hydrogen utilization and propionate formation [80]. Increased propionate flux is favorable for glucogenic energy supply, thereby supporting higher lactose synthesis and milk yield. Similarly, enrichment of *Lachnospira*, a known fiber-degrading genus producing acetate and butyrate, suggests improved hemicellulose utilization and energy harvesting from fibrous diets [82]. This provides a mechanistic explanation for improved feed efficiency observed in studies using MOS or EOB-based additives. By contrast, the reduction in *Enterobacter* and *Acinetobacter* may indicate suppression of opportunistic taxa often associated with inflammatory responses or metabolic stress in cattle [83,84] GEO supplementation increased *Macellibacteroides* and *Erysipelatoclostridium*. *Macellibacteroides*, a member of the phyla *Bacteriodetes*, degrades complex carbohydrates such as cellulose, xylan, and pectin, potentially enhancing fiber digestion and energy yield in ruminants [66,85]. Feed additives that suppress methanogens may shift fermentation pathways, increasing taxa like Erysipelatoclostridium, possibly as an adaptive response [86]. However, elevated Erysipelatoclostridium abundance has been associated with gut dysbiosis and inflammation, which could undermine the benefits of improved fermentation and negatively impact animal health [87]. This indicates the need for careful dose optimization of EOB-containing additives to maximize beneficial effects while minimizing unintended shifts toward pro-inflammatory taxa.

*Escherichia*–*Shigella* taxa are considered opportunistic pathogens, and their enrichment in the rumen or hindgut microbiome is often interpreted as a marker of dysbiosis, reduced VFA production, or increased risk of enteric disturbances. Elevated levels can signal dietary or health stress in cattle [88]. OLEO supplementation uniquely enriched *Christensenellaceae* and *Lysinibacillus*, both taxa with reported links to host metabolic health and fiber fermentation. *Christensenellaceae* are increasingly recognized as key contributors to gut homeostasis, with strong associations with efficient energy harvest and host metabolic regulation. Evidence from human and animal studies suggests that higher abundance of *Christensenellaceae* is linked to reduced risk of metabolic disorders such as obesity, diabetes, and inflammatory bowel disease. In addition, members of this family enhance microbial diversity and have been shown to stimulate the deconjugation of primary bile acids, including taurocholic acid, thereby influencing bile acid metabolism and overall gut functionality [89]. *Lysinibacillus* strains, including *L. fusiformis* and *L. sphaericus*, exhibit fibrolytic and probiotic properties by producing antimicrobial compounds, enhancing nutrient utilization, and supporting gut health. Their biocontrol and immunomodulatory activities suggest potential benefits in ruminants for improving fermentation efficiency, disease resistance, and overall productivity [90,91]. Suppression of *Vagococcus* and *Catenibacterium*, genera with limited beneficial roles in the rumen, may further stabilize fermentation profiles by reducing less efficient fermentative pathways [75]. Collectively, these microbial shifts suggest that prebiotics (MOS and/or GOS), EOB, and OPE mixture each create selective pressures that favor taxa linked to fiber degradation, VFA production, and reduced opportunistic growth. This indicates that such additive combinations could be strategically applied to enhance rumen function, reduce disease risk, and improve dairy performance while contributing to environmentally sustainable production. This targeted modulation of rumen microbiota supports improved fermentation efficiency, metabolic resilience, and ultimately dairy productivity.

The strong correlations between fibrolytic and fermentative taxa such as *Butyrivibrio*, *Lachnospiraceae_UCG-009*, *Macellibacteroides*, *Succinimonas*, and *Lachnospira* and phenolic derivatives, amino-acid catabolites, and indole-related metabolites indicate coordinated shifts in aromatic-compound metabolism and carbohydrate fermentation. Many of these metabolites (e.g., coniferyl acetic acid, pyrocatechol, indoxyl) arise from microbial transformation of plant phenolics and tryptophan, which support epithelial integrity, antioxidant defense, and immune modulation through pathways such as AhR and serotonergic signaling [42,43]. These correlations suggest that GEO, MEO, and OLEO enhance microbial utilization of phenolics while promoting VFA-producing taxa, thereby reinforcing rumen homeostasis and fermentation stability.

Positive associations between *Succinimonas*, *Prevotellaceae*, and creatine-, guanine-, or dihydroxyphenyl-related metabolites further imply strengthened energy metabolism, propionate formation, and microbial protein synthesis [79,80]. Concurrent correlations involving N(6)-methyllysine, indoxyl, and amino-fatty-acid intermediates highlight links to nitrogen turnover, methylation, and host immune-metabolic regulation [51]. Collectively, these relationships indicate that the additives promote a rumen environment favoring fibrolysis, propionate-directed hydrogen economy, reduced inflammatory amines, and improved nutrient assimilation.

## 5. Conclusions

This study demonstrates that GEO, MEO, and OLEO each differentially modulated rumen metabolism and microbial composition while maintaining stable fermentation performance. GEO primarily enhanced tryptophan, tyrosine, and purine metabolism, and MEO stimulated phosphonate and pyrimidine pathways alongside bile acid biosynthesis, while OLEO promoted phosphonate, nicotinamide, and taurine metabolism. At the microbial level, each additive enriched distinct taxa, including *Lachnospira*, *Succinivibrionaceae*, *Macellibacteroides*, *Lysinibacillus*, and *Christensenellaceae*, reflecting complementary modes of action. These changes collectively support improved amino acid and nucleotide flux, lipid utilization, antioxidant capacity, and microbial stability. The upregulation of phenolic and antioxidant metabolites, combined with reductions in histamine, further indicates enhanced rumen health. Importantly, these effects were achieved without compromising the overall balance of dominant microbial phyla, highlighting the safety and ecological compatibility of these additive combinations. Overall, the metabolic and microbial shifts suggest potential benefits for dairy cows through improved feed efficiency, resilience to metabolic stress, reduced methane emissions, and enhanced productivity. Future in vivo validation is warranted to confirm these RUSITEC-based findings under practical feeding conditions and to assess long-term impacts on animal health, milk yield, and environmental sustainability.

## Figures and Tables

**Table 1:** Differentially Abundant Metabolites Identified by Volcano Plot Analysis.

Group	Direction	Metabolite	Fold Change	Log2(FC)
GEO	Upregulated	Isomer 1 of Hulupinic acid	3.79	1.92
	Upregulated	2-Aminomuconic acid	2.52	1.34
	Upregulated	4-Hydroxyphenylethanol	2.40	1.27
	Upregulated	Coniferyl acetic acid	2.28	1.19
	Upregulated	5-Hydroxyindoleacetic acid	2.27	1.19
	Upregulated	Isomer 2 of 3,4-Dihydroxyphenylvaleric acid	2.14	1.10
	Downregulated	Dopamine 4-O-glucuronide	0.29	−1.80
	Downregulated	Isomer 1 of 2-Hydroxyhepta-2,4-dienedioic acid	0.31	−1.67
	Downregulated	Histamine	0.34	−1.55
	Downregulated	Chlorohydroquinone	0.36	−1.48
	Downregulated	Tryptophanamide	0.49	−1.03
MEO	Upregulated	5-Hydroxyindoleacetic acid	2.41	1.27
	Upregulated	Dihydropinosylvin	2.37	1.24
	Upregulated	Pyrocatechol	2.35	1.23
	Upregulated	N-Carboxyethyl-g-aminobutyric acid	2.33	1.22
	Upregulated	Eugenitin	2.20	1.14
	Upregulated	Piceatannol	2.17	1.12
	Upregulated	Dulciol E	2.14	1.09
	Upregulated	2-Phenylethylamine	2.10	1.07
	Downregulated	Dopamine 4-O-glucuronide	0.30	−1.75
	Downregulated	Histamine	0.31	−1.70
	Downregulated	Isomer 1 of 2-Hydroxyhepta-2,4-dienedioic acid	0.37	−1.42
	Downregulated	Cis-Indole-2,3-Dihydrodiol	0.40	−1.34
	Downregulated	Norfuraneol	0.41	−1.29
	Downregulated	2-Hydroxy-6-oxonona-2,4-diene-1,9-dioic acid	0.43	−1.20
	Downregulated	Aminoacrylic acid	0.44	−1.18
	Downregulated	Aspartyl-Glycine	0.46	−1.12
OLEO	Upregulated	2,3,6-Trihydroxypyridine	3.41	1.77
	Upregulated	N(6)-Methyllysine	2.22	1.15
	Upregulated	Eugenol	2.19	1.13
	Upregulated	Phenylpropanolamine	1.98	0.98
	Upregulated	Prolyl-proline	1.97	0.98
	Upregulated	Taurine	1.94	0.96
	Upregulated	N-Carboxyethyl-g-aminobutyric acid	1.94	0.95
	Downregulated	Chlorohydroquinone	0.50	−1.00
	Downregulated	Aspartyl-Aspartate	0.47	−1.09
	Downregulated	Choline	0.42	−1.26
	Downregulated	Tryptophanamide	0.37	−1.44
	Downregulated	Histamine	0.31	−1.70

GEO received GOS, EOB and OPE; MEO received MOS, EOB and OPE; OLEO received MOS, GOS, EOB and OPE.

**Table 2:** Effect of combined prebiotics (GOS, MOS), EOB and OPE mixture on ruminal microbial composition.

Treatments	Bacteria, OTUs	Archaea, OTUs	Unassigned, OTUs
CON	528,844	12,124	0.00
GEO	436,329	9970	0.00
MEO	520,188	10,316	1.25
OLEO	471,850	9589	0.00
Pooled SEM	39,983	1461	0.625
*p*-value	0.361	0.633	0.426

**Table 3:** Effect of combined prebiotics (GOS, MOS), EOB and OPE on ruminal microbial abundance.

	Treatments				SEM	*p*-Value
	CON	GEO	MEO	OLEO		
d__Bacteria; p__*Firmicutes*	270,209	241,990	300,312	245,491	20,916	0.227
d__Bacteria; p__*Proteobacteria*	128,746	77,001	79,447	89,466	23,131	0.396
d__Bacteria; p__*Bacteroidota*	80,499	72,088	97,175	78,761	8387	0.237
d__Bacteria; p__*Actinobacteriota*	10,550	8761	11,608	8806	1418	0.440
d__Bacteria; p__*Campilobacterota*	9464	10,424	2936	21,430	5762	0.206
d__Bacteria; p__*Desulfobacterota*	8871	7540	8394	7393	1844	0.931
d__Bacteria; p__*Spirochaetota*	8624	7136	7098	8559	2469	0.947
d__Bacteria; p__*Fibrobacterota*	3021	2776	3025	3473	807	0.941
d__Bacteria; p__*Synergistota*	1379	1242	1278	1076	199	0.755
d__Bacteria; p__*Chloroflexi*	1264	1453	1683	1132	270	0.525
d__Bacteria; p__*Elusimicrobiota*	789	1113	681	770	145	0.219
d__Bacteria; p__*Patescibacteria*	2062	1781	2655	1866	361	0.355
d__Bacteria; p__*Planctomycetota*	2183	1884	1810	1971	298	0.830
d__Bacteria; p__*Verrucomicrobiota*	889	781	1658	1317	460	0.532
d__Bacteria; p__*Bdellovibrionota*	177	254	237	189	93.3	0.925
d__Bacteria; p__SAR324_*clade*(Marine_group_B)	62.0	44.5	129.8	87.8	25.7	0.157
d__Bacteria; p__WPS-2	16.5	18.0	13.3	23.0	10.9	0.935
d__Bacteria; p__*Cyanobacteria*	16.8	27.0	32.3	26.8	13.6	0.875
d__Bacteria; p__*Myxococcota*	13.8	10.3	9.75	12.3	4.51	0.916
d__Bacteria; __	7.75	6.75	6.75	2.25	3.01	0.587
d__Bacteria; p__*Fusobacteriota*	0.50	0.00	0.00	0.00	0.25	0.426
d__Bacteria; p__*Deferribacterota*	0.00	0.00	0.50	0.00	0.25	0.426
d__Archaea; p__*Euryarchaeota*	11,799	9667	10,085	9341	1421	0.632
d__Archaea; p__*Thermoplasmatota*	324	303	230	246	65.1	0.701
d__Archaea; p__*Halobacterota*	0.75	0.00	0.75	2.50	0.98	0.365
Unassigned; __	0.00	0.00	1.25	0.00	0.63	0.426

**Table 4:** Differential abundance of all taxa between MEO and CON at the genus level.

	Log2FC	Std. Error	*p*-Value
*Pseudarcobacter*	3.47	1.450	0.0340
*Lachnospira*	2.68	0.469	1.0 × 10^−4^
*Tyzzerella*	2.58	0.482	1.8 × 10^−4^
*Succinivibrionaceae*	1.89	0.616	0.0097
*Lineage_IV*	1.72	0.744	0.0391
*Eubacterium_ventriosum_group*	1.68	0.405	0.0013
*Cellulosilyticum*	1.59	0.761	0.0587
*UCG_001*	1.42	0.444	0.0076
*Brevundimonas*	1.39	0.616	0.0441
*Termite_Treponema_cluster*	1.27	0.473	0.0196
*Prevotellaceae_UCG_003*	1.26	0.367	0.0050
*Aeromicrobium*	1.03	0.448	0.0401
*RF39*	1.01	0.423	0.0338
*Acinetobacter*	−2.84	0.812	0.0044
*f__Rhodobacteraceae*	−2.61	0.808	0.0073
*Enterobacter*	−2.21	0.824	0.0197
*Agathobacter*	−2.12	0.616	0.0049
*Comamonas*	−2.10	0.838	0.0277
*Enterococcus*	−1.88	0.622	0.0105
*uncultured_19*	−1.60	0.643	0.0288
*Desemzia*	−1.40	0.525	0.0202

**Table 5:** Differential abundance of all taxa between GEO and CON at the genus level.

	Log2FC	Std. Error	*p*-Value
*Uncultured_1*	10.9	1.420	5.93 × 10^−6^
*Incertae_Sedis*	6.20	0.787	4.44 × 10^−6^
*Macellibacteroides*	5.72	0.892	3.34 × 10^−5^
*Erysipelatoclostridium*	4.55	1.210	0.0028
*Lachnospiraceae_UCG_009*	3.05	0.606	3.0 × 10^−4^
*WPS_2*	2.40	2.050	0.2630
*Escherichia_Shigella*	2.39	1.070	0.0456
*Tyzzerella*	2.16	0.482	8.0 × 10^−4^
*uncultured_12*	1.89	0.787	0.0331
*Butyrivibrio*	1.18	0.257	6.0 × 10^−4^
*V9D2013_group*	1.17	0.507	0.0394
*Endomicrobium*	1.01	0.392	0.0240
*Enterococcus*	−2.79	0.622	7.0 × 10^−6^
*Tuzzerella*	−2.30	0.793	0.0134
*Agathobacter*	−2.27	0.616	0.0031
*Micrococcus*	−2.15	0.534	0.0017
*f__Prevotellaceae*	−1.87	0.460	0.0016
*f__Muribaculaceae*	−1.80	0.772	0.0377
*Desemzia*	−1.33	0.525	0.0264

**Table 6:** Differential abundance of all taxa between OLEO and CON at the genus level.

	Log2FC	Std. Error	*p*-Value
*Uncultured_1*	6.76	1.420	4.67 × 10^−4^
*Incertae_Sedis*	5.29	0.787	2.14 × 10^−5^
*Lysinibacillus*	4.59	1.580	0.0134
*Christensenellaceae*	3.65	1.250	0.0129
*Lachnospira*	3.45	0.469	8.87 × 10^−6^
*Lachnospiraceae_UCG_009*	2.00	0.606	6.42 × 10^−3^
*Tyzzerella*	1.79	0.482	2.99 × 10^−3^
*Cellulosilyticum*	1.69	0.761	0.0468
*Succinivibrionaceae_UCG_002*	1.60	0.723	0.0467
*Termite_Treponema_cluster*	1.39	0.473	0.0124
*UCG_001*	1.29	0.444	0.0133
*Succinimonas*	1.16	0.360	7.42 × 10^−6^
*Clostridium_sensu_stricto_1*	1.08	0.401	0.0198
*Enterococcus*	−2.83	0.622	6.76 × 10^−4^
*Vagococcus*	−2.65	0.900	0.0124
*Agathobacter*	−2.13	0.616	4.76 × 10^−3^
*Catenibacterium*	−2.13	0.885	0.0329
*Enterobacter*	−2.03	0.824	0.0300
*Uncultured_19*	−1.75	0.643	0.0186
*Desemzia*	−1.37	0.525	0.0227
*Mogibacterium*	−1.08	0.309	4.39 × 10^−3^
*f__Prevotellaceae*	−1.07	0.460	0.0382

## Data Availability

Data are contained within the article. Supplementary Data are also available in the repository.

## Supplementary Materials

The following supporting information can be downloaded at: https://www.mdpi.com/article/10.3390/metabo15120762/s1, Figures S1–S3: Box plot of samples of the metabolome between GEO, MEO, OLEO and CON groups before- and after- normalization; Figures S4–S6: Fold Change in the metabolites between GEO, MEO, OLEO and CON groups. Figures S7–S9: Permutation test showing the empirical values for both Q^2^ and R^2^Y for GEO, MEO and OLEO groups; Figures S10–S12: PLS-DA VIP scores show the metabolome between CON and GEO, MEO and OLEO groups; Figures S13–S15: Receiver–operator characteristic curves showing are biomarkers enhanced by GEO, MEO and OLEO treatments; Figure S16: Beta diversity of microbiota between the control and treatment groups; Figure S17: Effects of GEO, MEO and OLEO on the abundance of microbial taxa at the phylum level.; Files S1 (Excel file): PREOBOPE Metabolome supplementary showing results of PLS-DA VIP Scores, fold change, and volcano plot; Data on goods coverage, normalization library sizes, taxonomic abundance, and the results of the integrative microbiome–metabolome correlation analysis.
