# Supplementary material for: Rumen Fluid Metabolomics and Microbiome Profiling of Dairy Cows Fed Combinations of Prebiotics, Essential Oil Blend, and Onion Peel Using the RUSITEC System
Source: Metabolites. 2025 Nov 25;15(12):762. doi: 10.3390/metabo15120762 (PMC12734747; doi:10.3390/metabo15120762)
Supplement: Supplementary file 1 [file metabolites-15-00762-s001.zip › metabolites-3947927-supplementary.pdf]

Rumen fluid metabolomics of dairy cows fed combinations of prebiotics, essential oil blend, and onion peel using the RUSITEC system

__Supplementary Results__

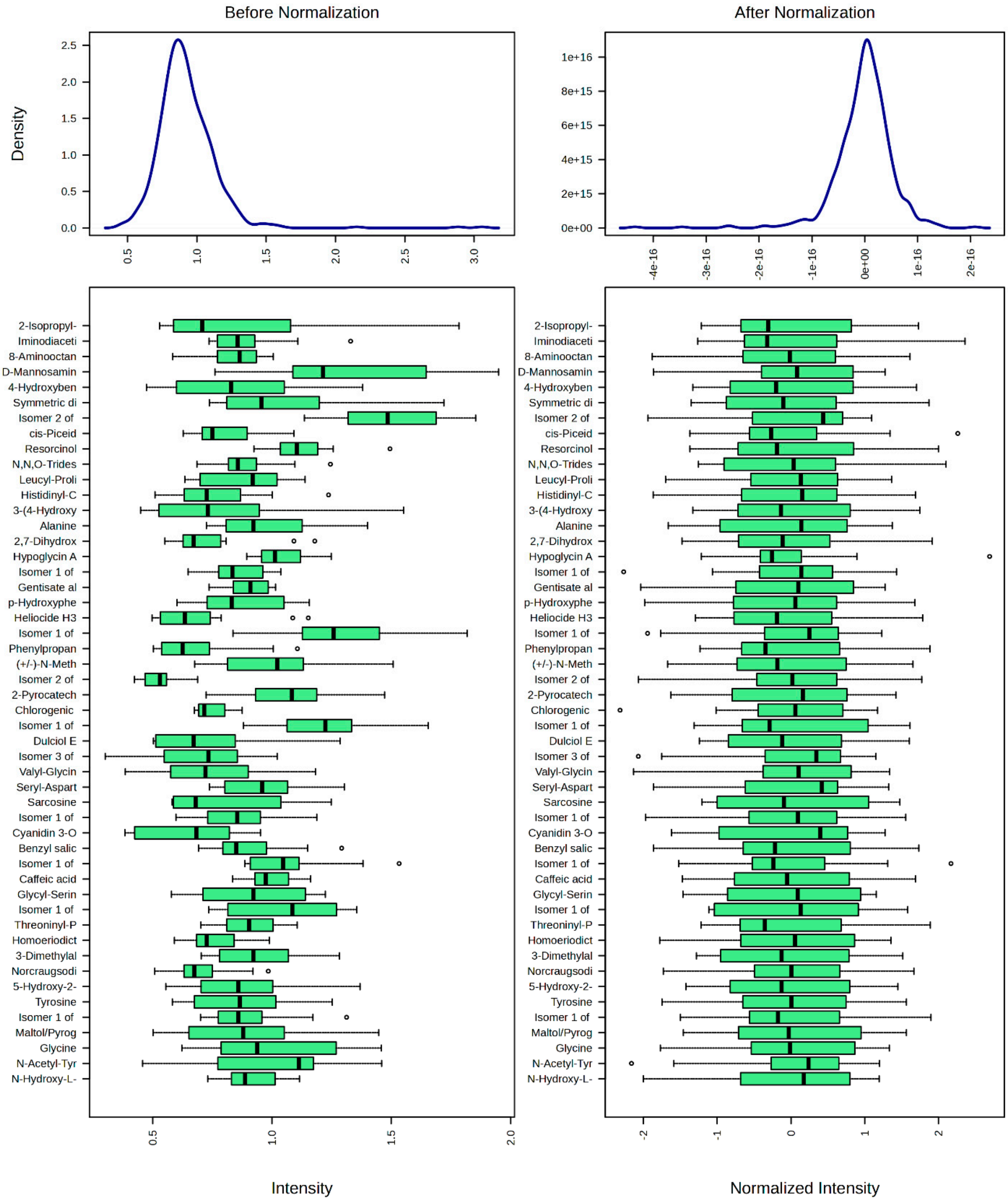

Figure S1. Box plot of samples of the metabolome between GEO and CON group before- and after-normalization.

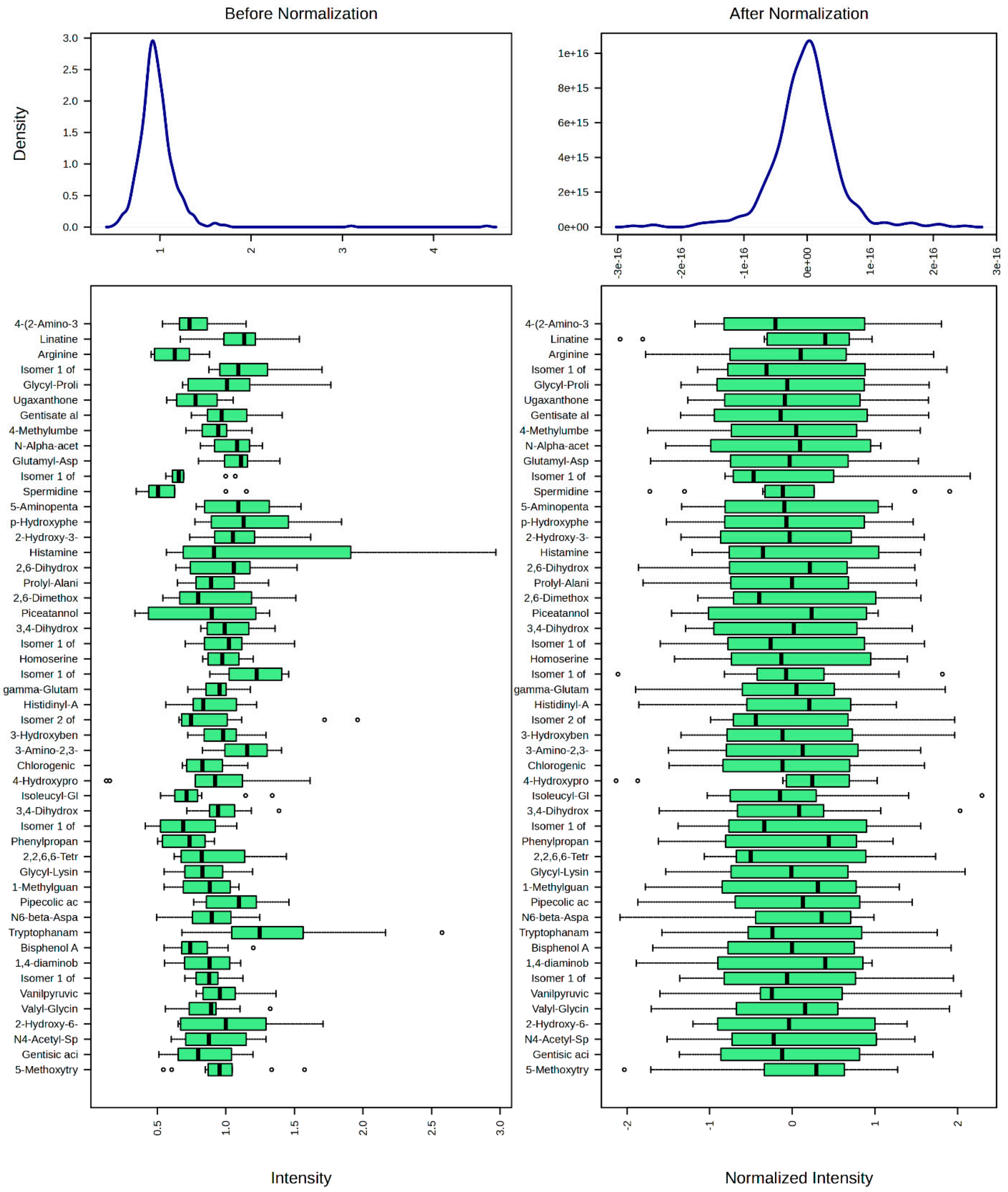

Figure S2. Box plot of samples of the metabolome between MEO and CON group before- and after-normalization.

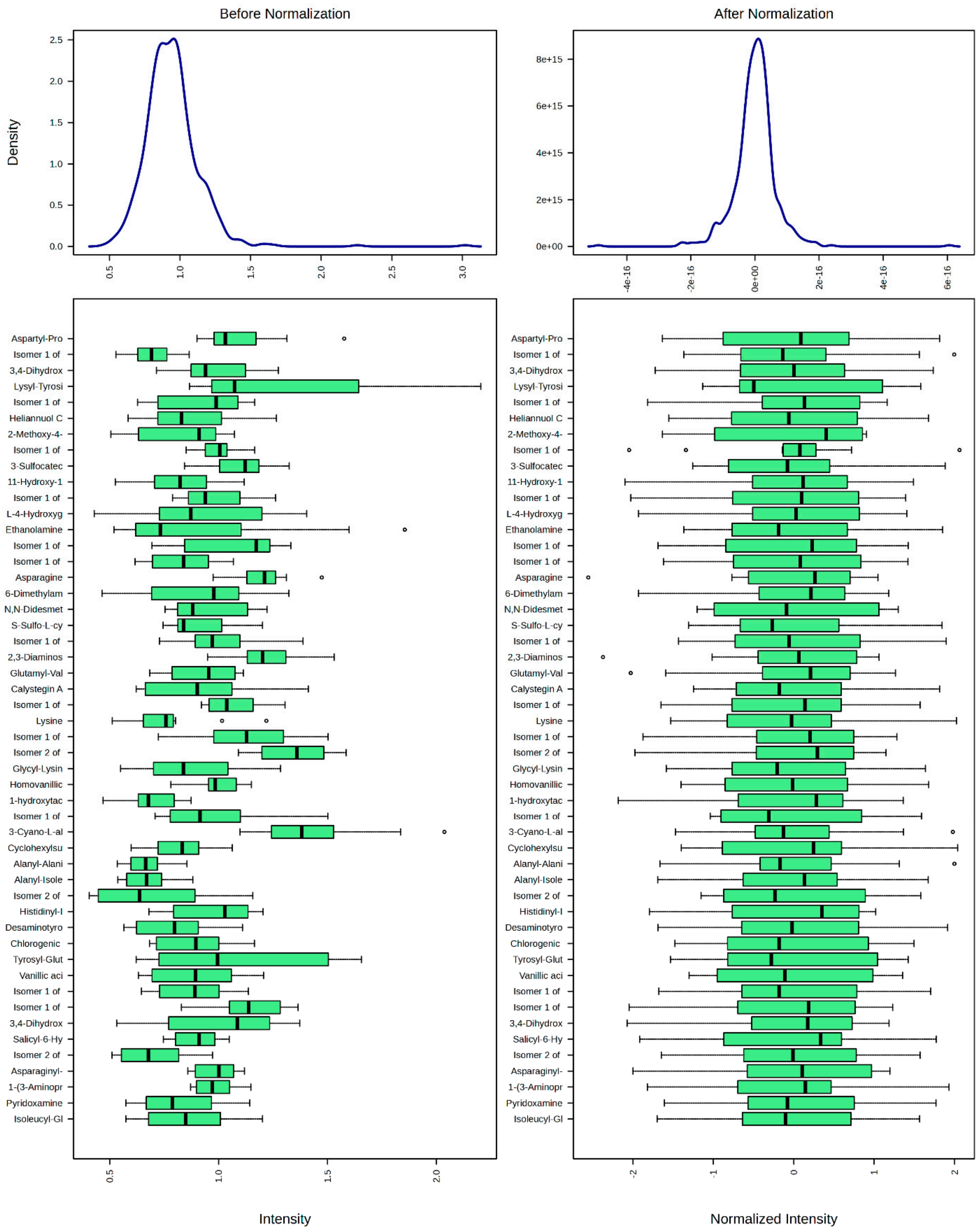

Figure S3. Box plot of samples of the metabolome between OLEO and CON group before- and after-normalization.

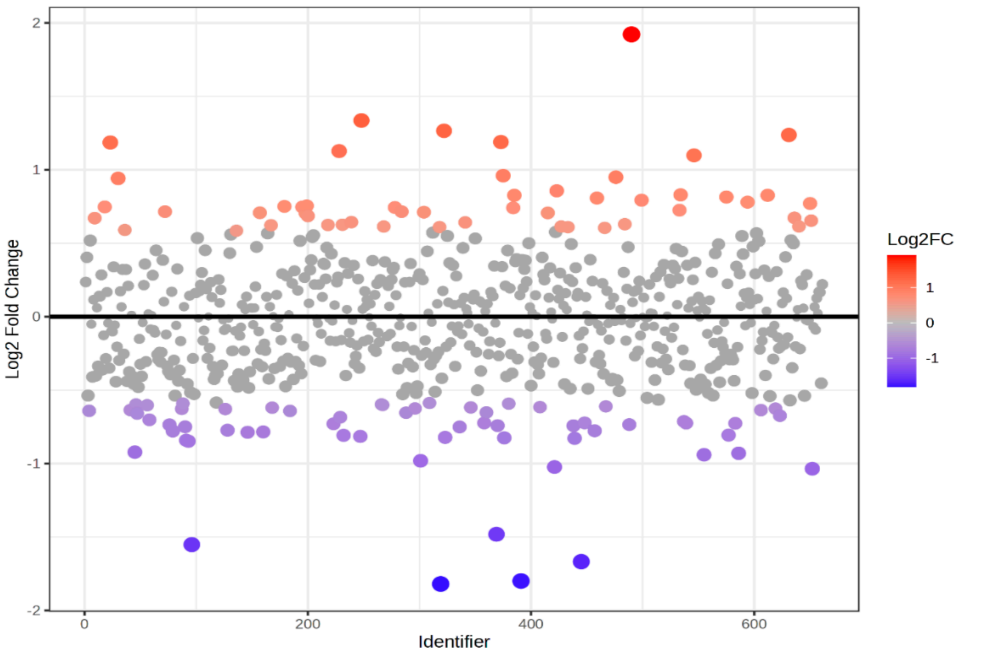

Figure S4. Fold Change of the metabolites between GEO and CON group.

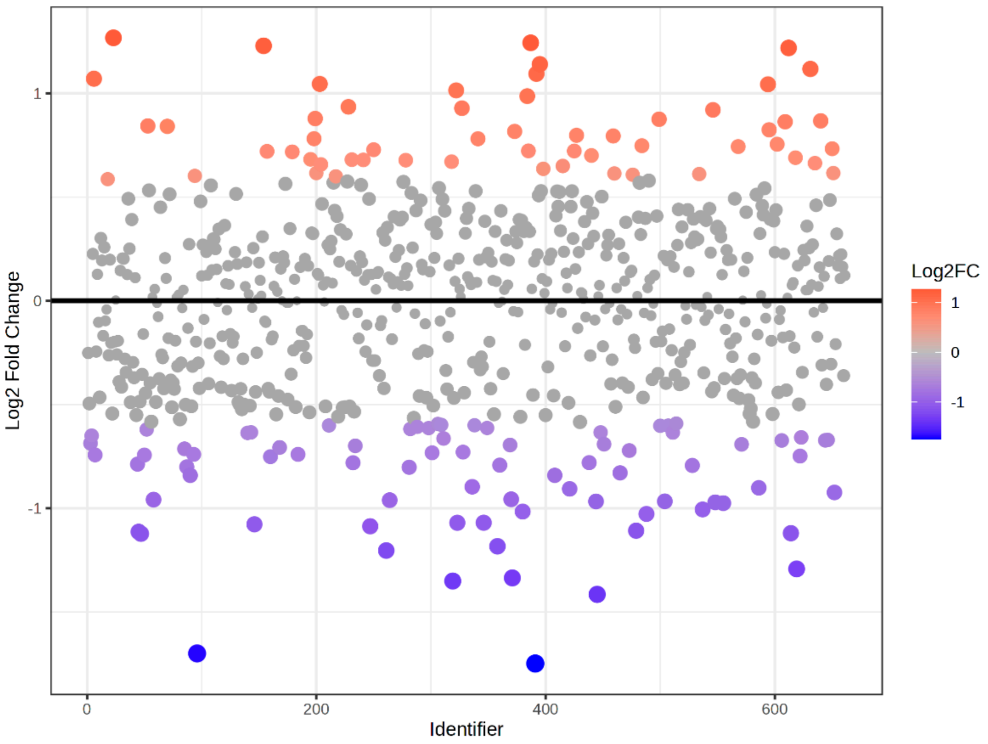

Figure S5. Fold Change of the metabolites between MEO and CON group.

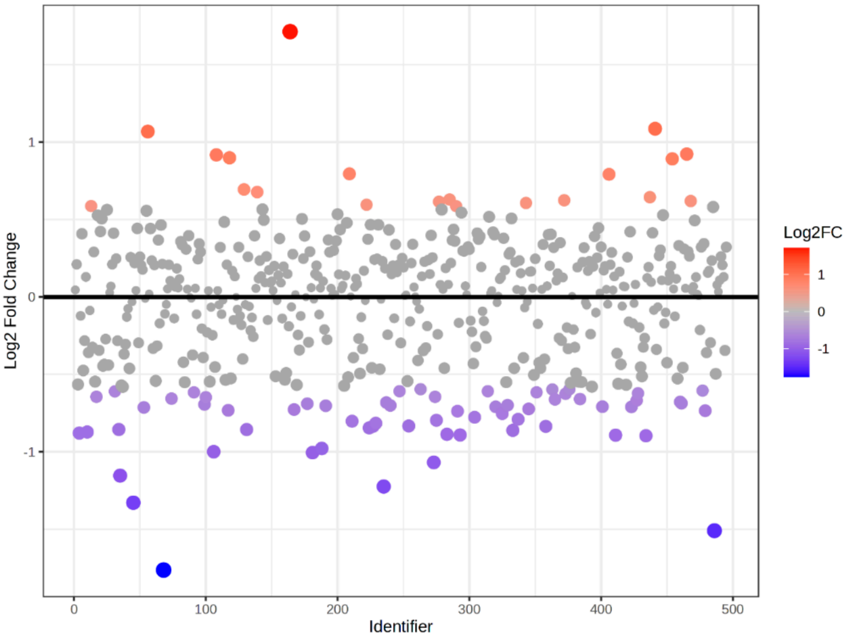

Figure S6. Fold Change of the metabolites between OLEO and CON group.

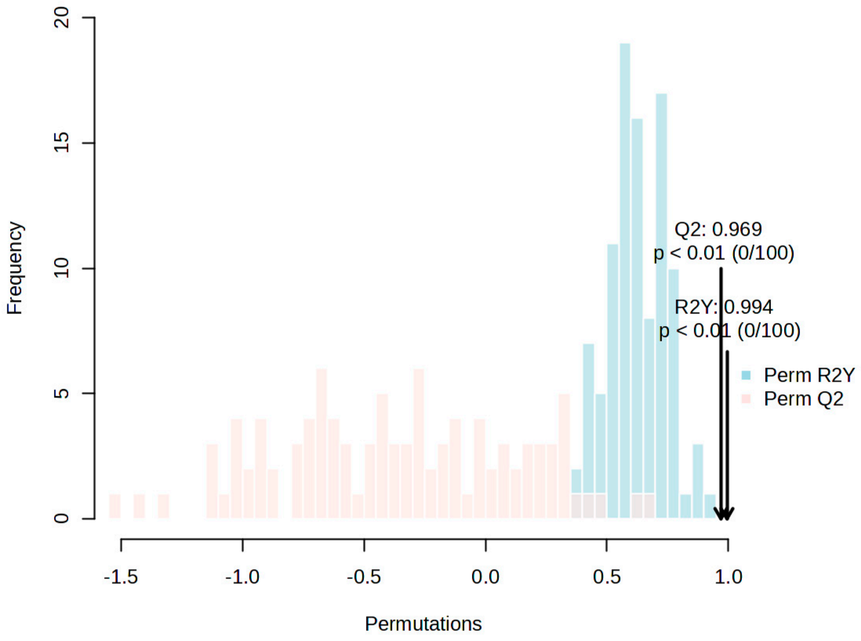

Figure S7. Permutation test showing the emperical values for both Q^2 ($p < 0.01$) and R^2Y ($p < 0.01$) for GEO group.

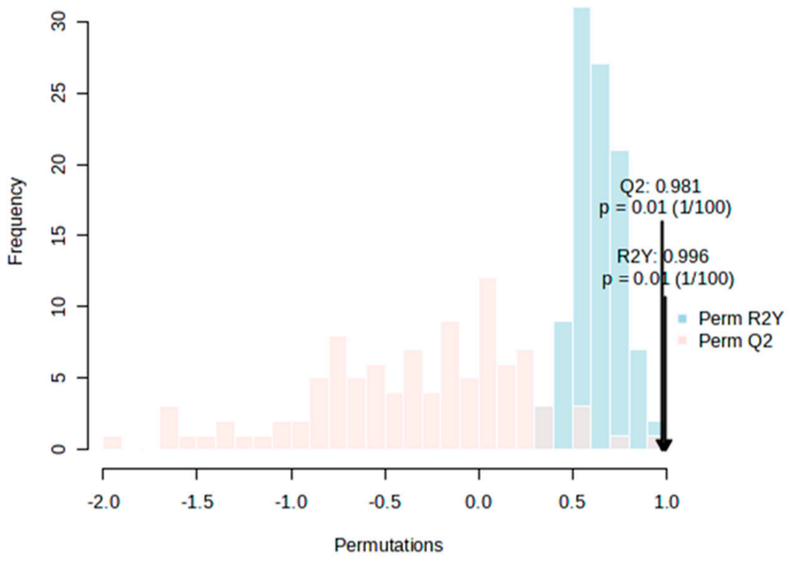

Figure S8. Permutation test showing the emperical values for both Q^2 ($p = 0.01$) and R^2Y ($p = 0.01$) for MEO group.

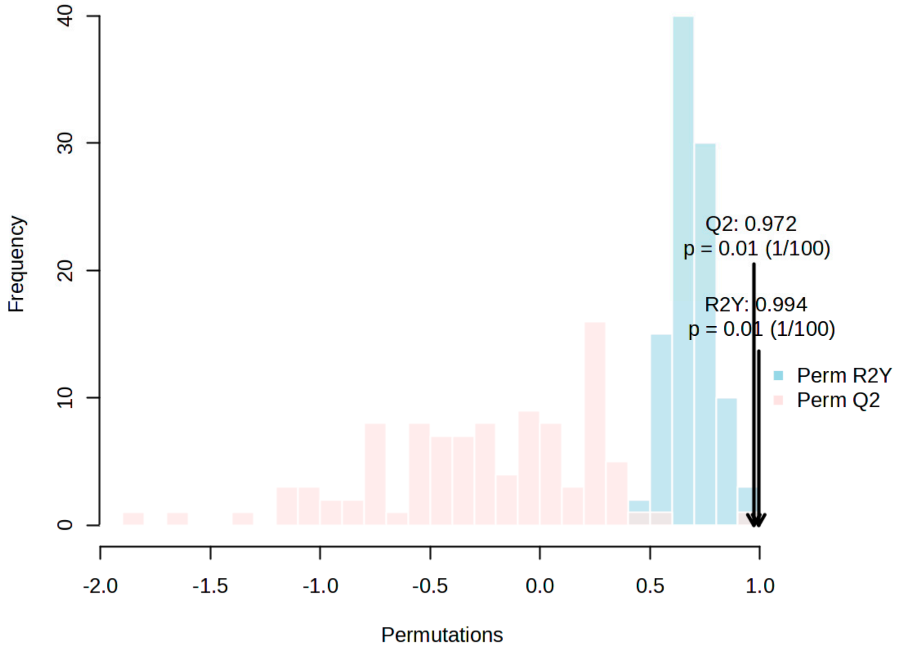

Figure S9. Permutation test showing the emperical values for both Q^2 ($p = 0.01$) and R^2Y ($p = 0.01$) for OLEO group.

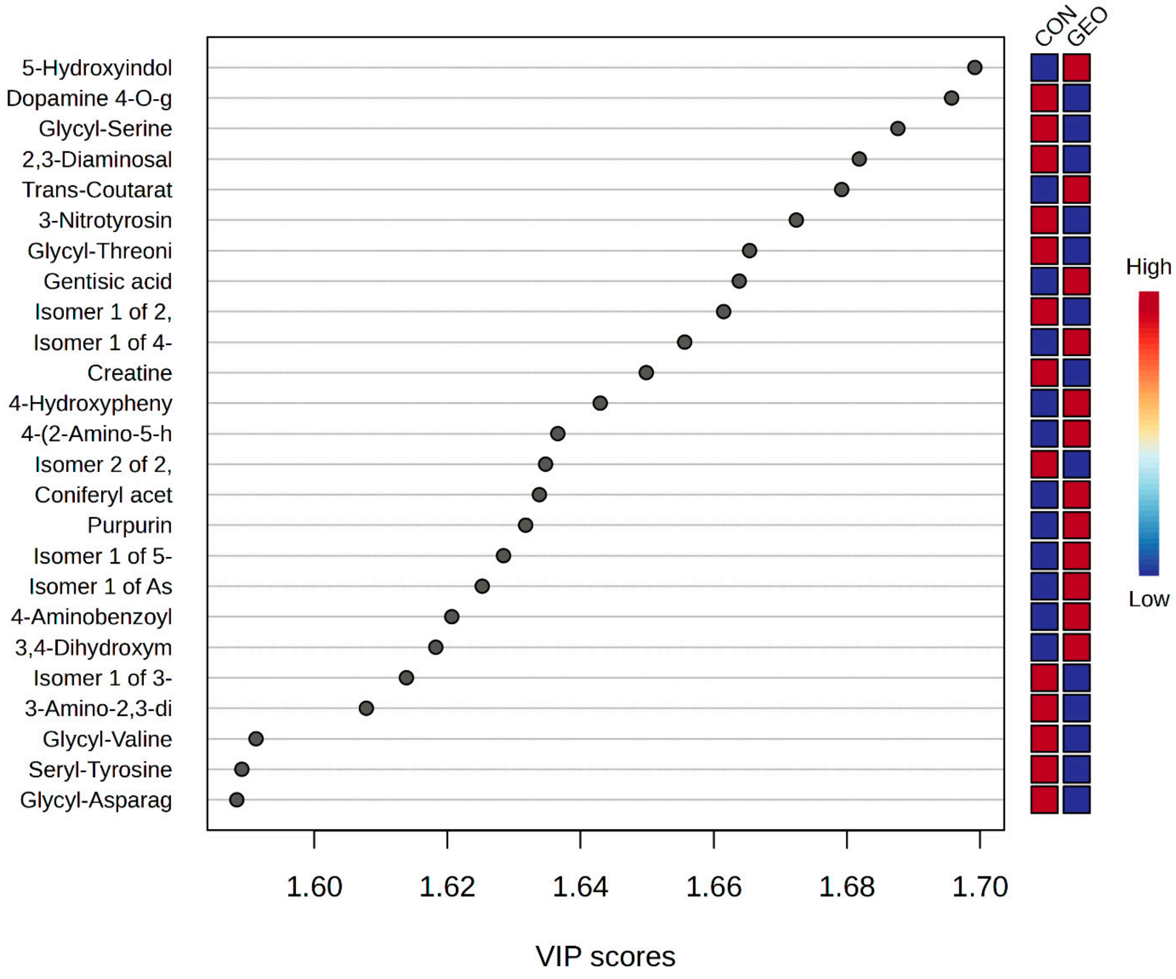

Figure S10. PLS-DA VIP scores show the metabolome between CON and GEO group.

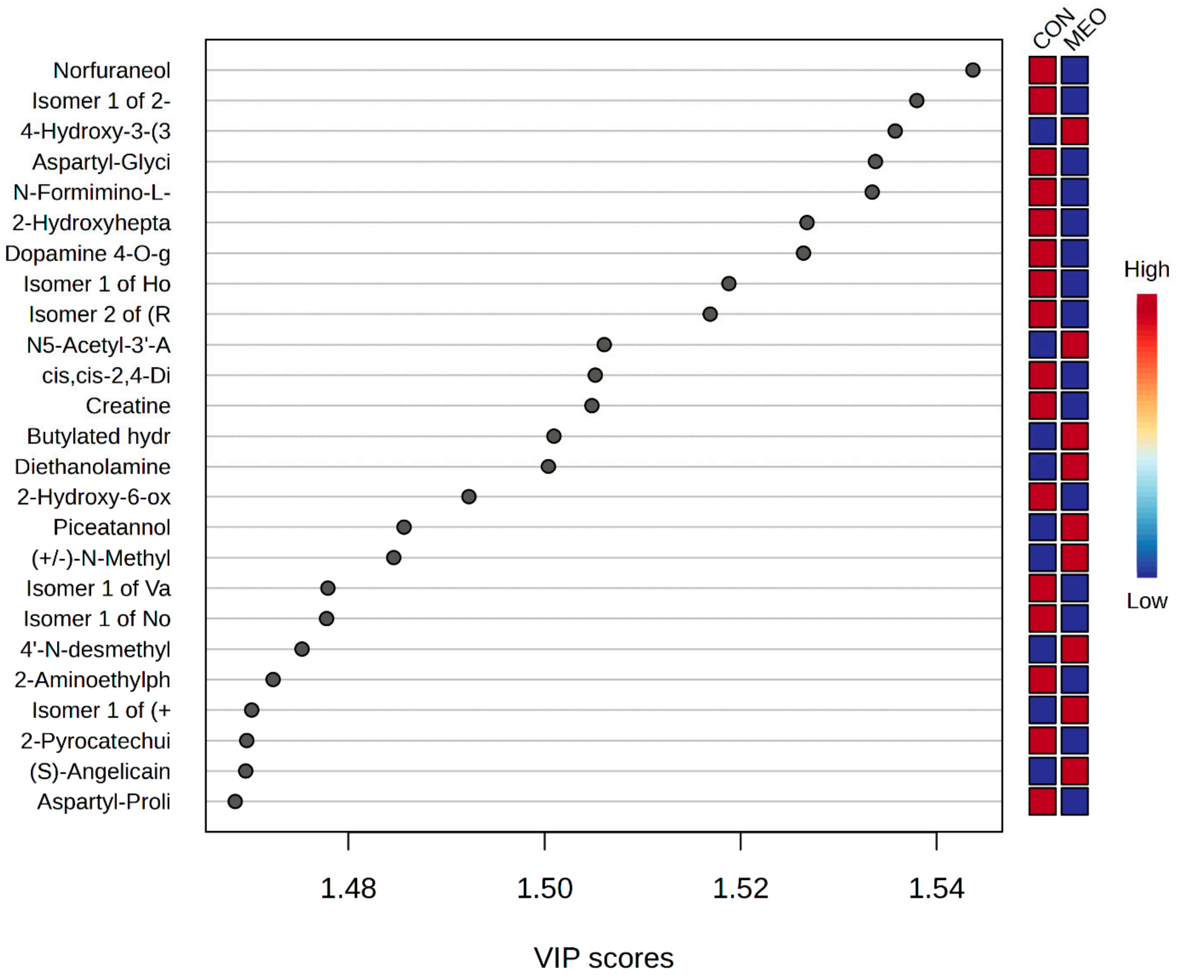

Figure S11. PLS-DA VIP scores show the metabolome between CON and MEO group.

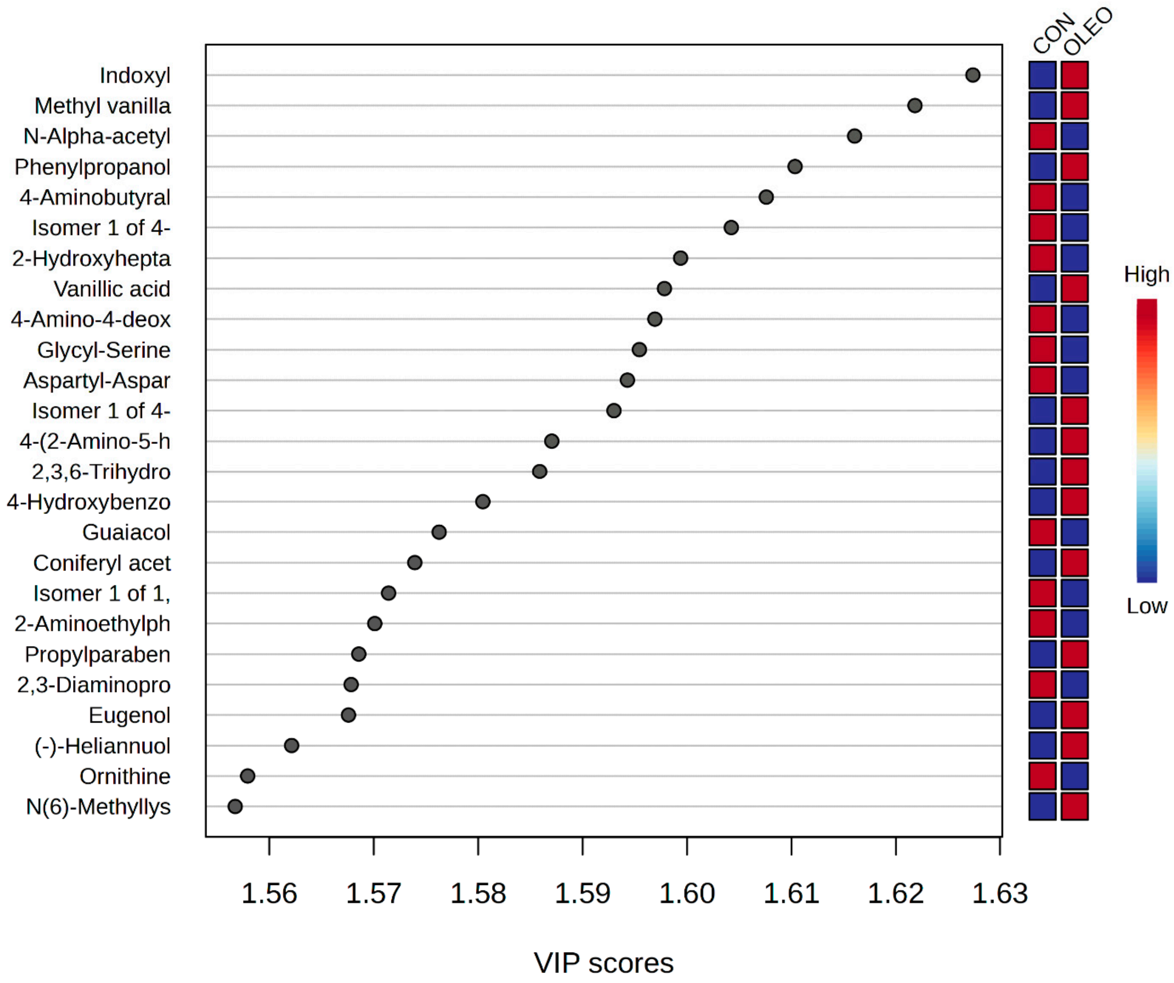

Figure S12. PLS-DA VIP scores show the metabolome between CON and OLEO group.

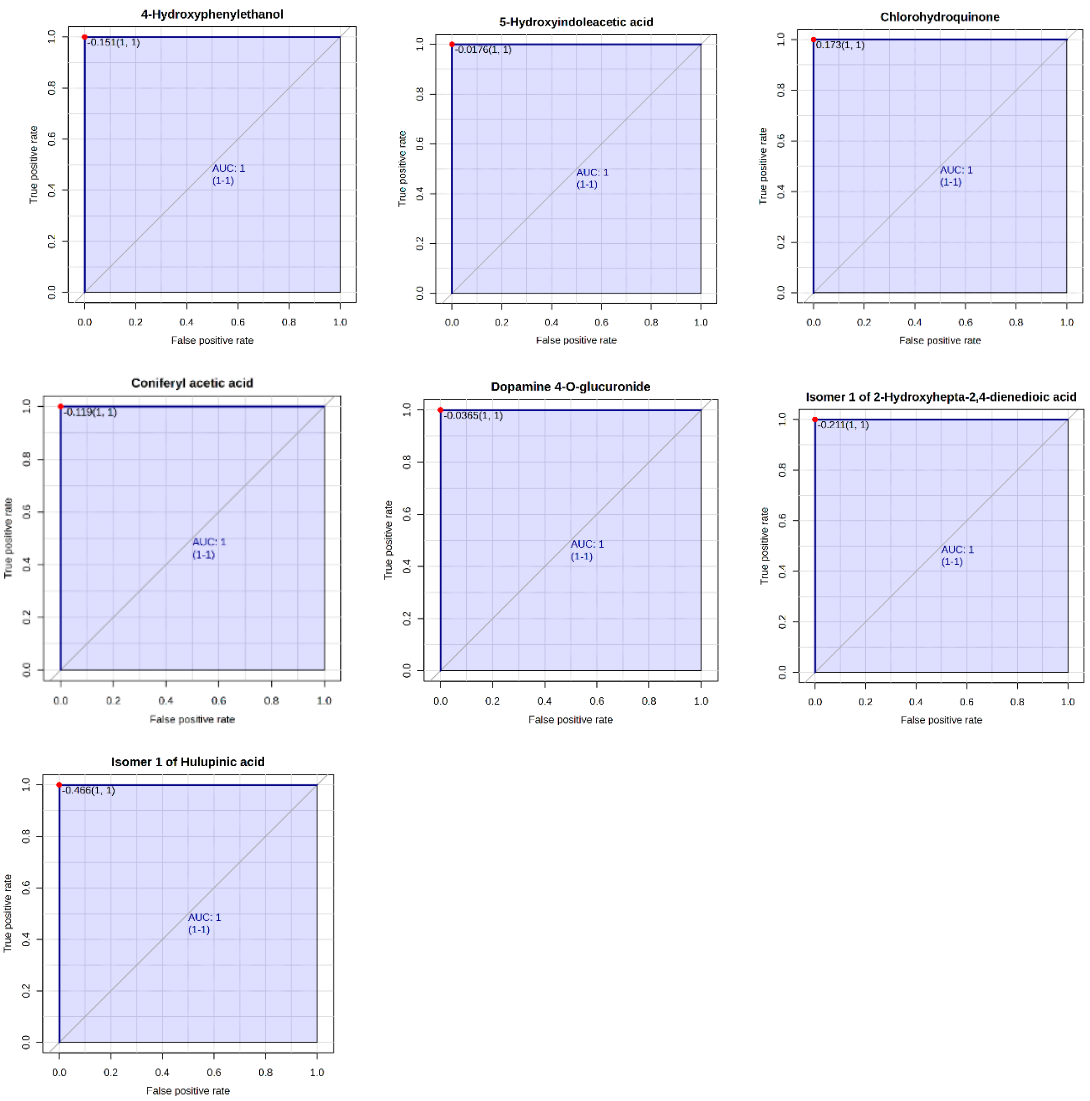

Figure S13. Receiver–operator characteristic curves of 4-Hydroxyphenylethanol, 5-Hydroxyindoleacetic acid, Chlorohydroquinone, Coniferyl acetic acid, Dopamine 4-O-glucuronide, Isomer 1 of 2-Hydroxyhepta-2,4-dienedioic acid, and Isomer 1 of Hulupinic acid which are biomarkers enhanced by GEO.

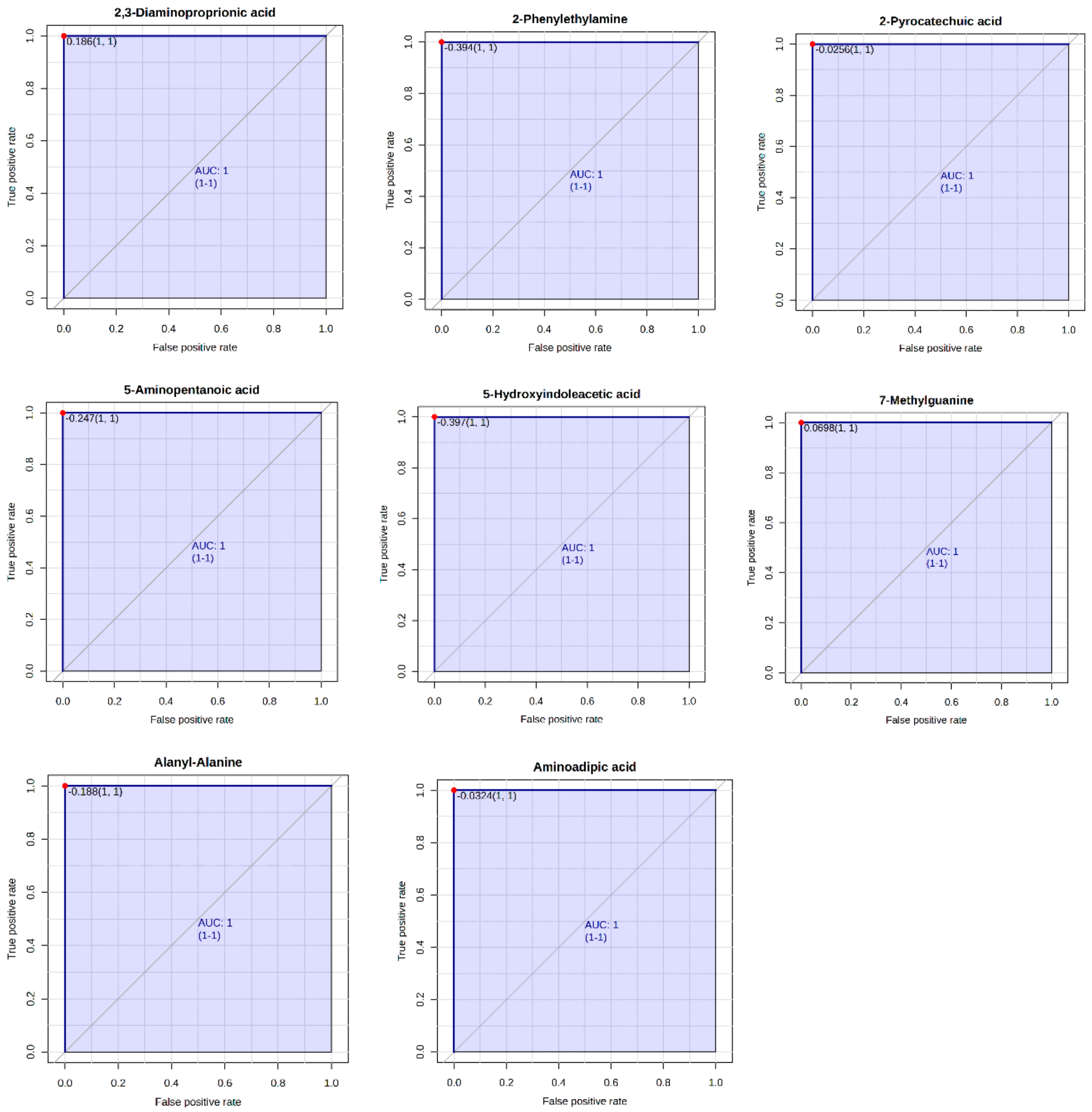

Figure S14. Receiver–operator characteristic curves of 2,3-Diaminoproprionic acid, 2-Phenylethylamine, 8-Aminooctanoic acid, 2-Pyrocatechuic acid, 5-Aminopentanoic acid, 5-Hydroxyindoleacetic acid, 7-Methylguanine, Alanyl-Alanine, and Aminoadipic acid which are biomarkers enhanced by MEO.

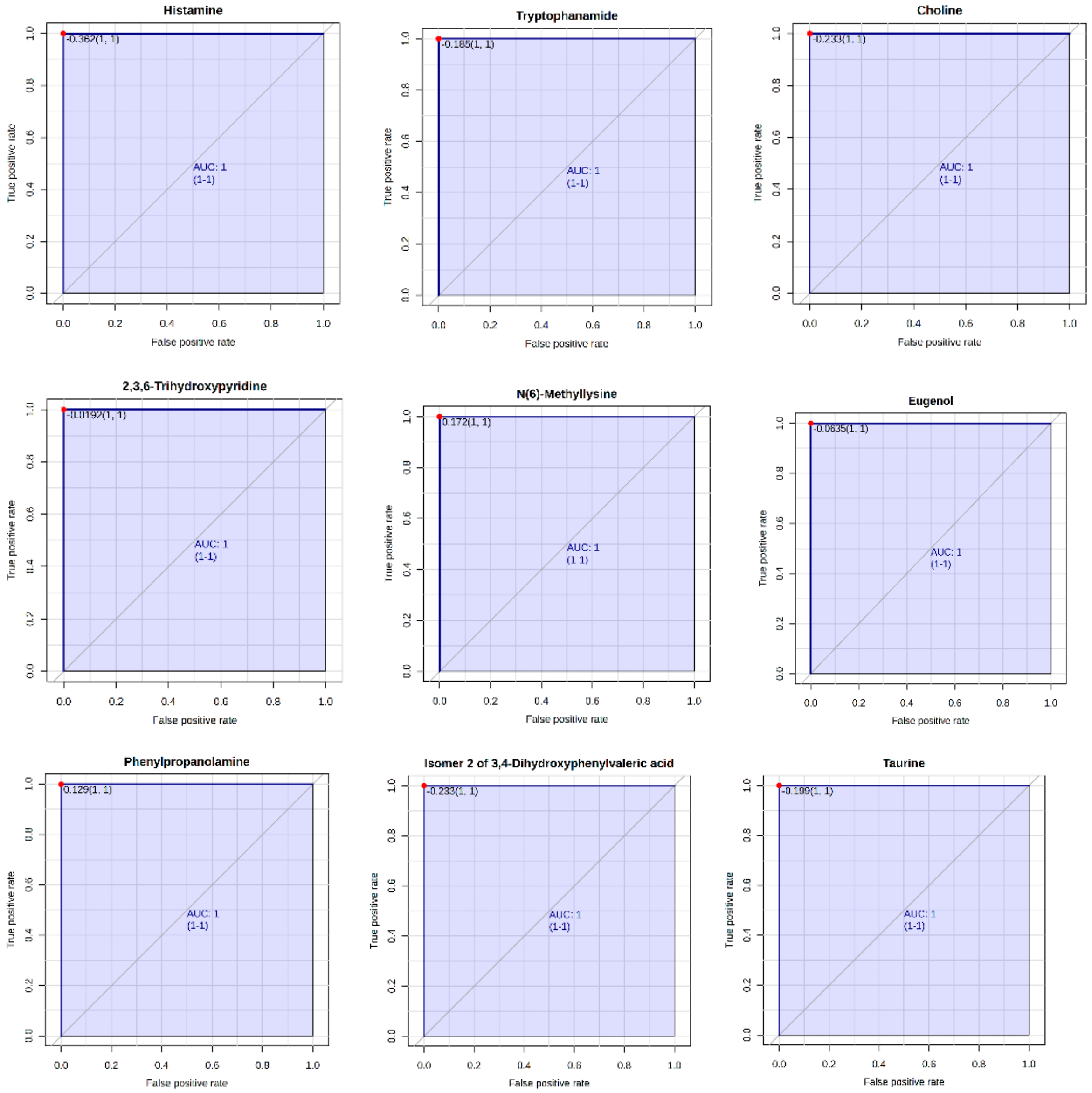

Figure S15. Receiver–operator characteristic curves of Histamine, Tryptophanamide, Choline, 2,3,6-Trihydroxypyridine, N(6)-Methyllysine, Eugenol, Phenylpropanolamine, Isomer 2 of 3,4-Dihydroxyphenylvaleric acid and Taurine which are biomarkers enhanced by OLEO.

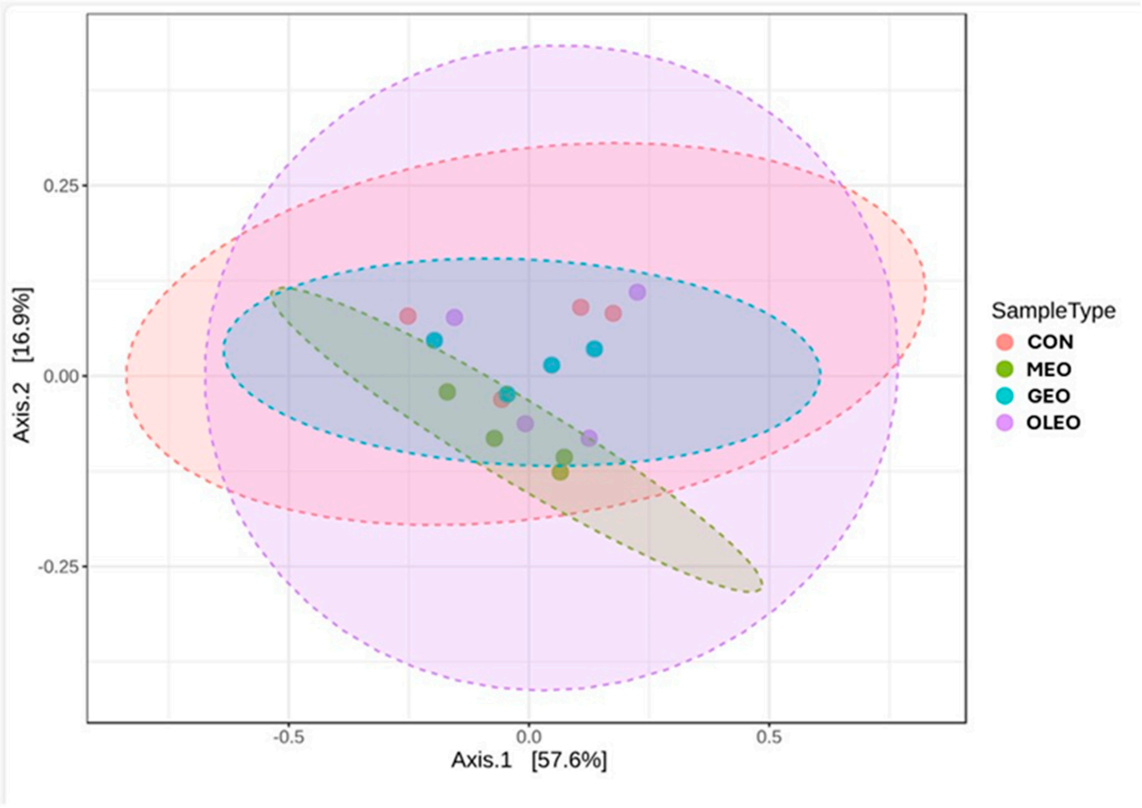

Figure S16: Beta diversity shows no clear separation between the control and treatment groups.

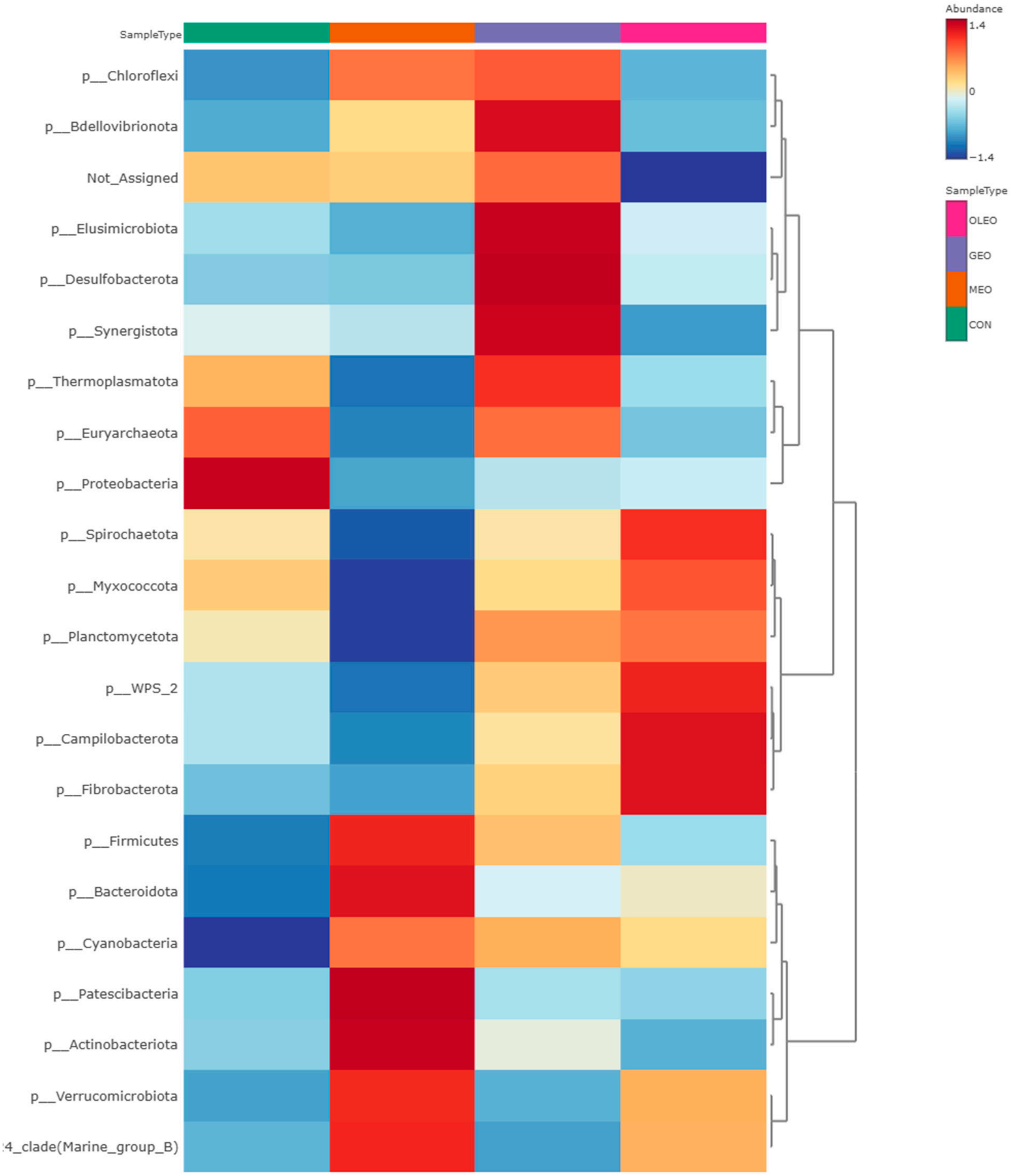

Figure S17: Effects of GEO, MEO and OLEO on the abundance of microbial taxa at the phylum level.
